# Material‐Dependent Functionalization of CVD‐Grown TMDC Monolayers Probed by Vibrational Nanospectroscopy

**DOI:** 10.1002/smll.74424

**Published:** 2026-07-06

**Authors:** Maziar Jafari, Amir Khojastehnezhad, Seyed Faridedin Rafie, Andrey Krayev, Nidal Abu‐Zahra, Mohamed Siaj

**Affiliations:** ^1^ Department of Chemistry Université Du Québec à Montréal (UQAM) Montréal Québec Canada; ^2^ Department of Chemical Engineering and Biotechnological Engineering, Faculty of Engineering Université De Sherbrooke Sherbrooke Quebec Canada; ^3^ Materials Science and Engineering Department University of Wisconsin‐Milwaukee Milwaukee Wisconsin USA; ^4^ HORIBA Scientific 359 Bel Marin Keys Blvd. Novato USA

**Keywords:** covalent surface functionalization, interfacial molecular dynamics, material‐specific reactivity, two‐dimensional (2D) materials, vibrational nanospectroscopy imaging

## Abstract

Understanding the molecular‐level mechanisms governing covalent functionalization of two‐dimensional semiconductors remains a major challenge. Here, we investigate the chemical vapor deposition (CVD) growth of monolayer MoSe_2_, WSe_2_, and MoSe_2_‐WSe_2_ in‐plane heterostructures, which were subsequently transferred onto conductive Au(111) substrates using a contamination‐ and defect‐free protocol. Tip‐enhanced Raman spectroscopy (TERS) and photo‐induced force microscopy (PiFM) are high‐resolution scanning probe techniques, enabling colocalized topographical and vibrational spectroscopic mapping of surface structures and bound‐molecules, providing sub‐diffraction of light spatial resolutions. Using TERS, we provide unique Raman nanospectroscopy analyses and imaging of lateral and vertical‐lateral MoSe_2_‐WSe_2_ nanodomains and alloyed heterojunctions. Applying PiFM, we directly visualize electrodeposited aryl diazonium salts, providing key direct nanoscale chemical evidence of 4‐carboxyphenyl (4‐CP) and 4‐nitrophenyl (4‐NP) covalent functionalization on transition metal dichalcogenides (TMDCs) and their respective heterostructures in protic and organic solvents, revealing material‐dependent reactivity. Atomistic molecular dynamics simulations reveal that 4‐nitrobenzenediazonium cations (4‐NBD^+^) interact more strongly with MoSe_2_ than WSe_2_, exhibiting persistent interfacial hydration and reduced mobility, which localizes precursors at the interface. This sustained interfacial availability rationalizes the experimentally observed higher reduction probability and grafting efficiency on MoSe_2_. Together, these findings provide a robust molecular‐level framework for solvent‐ and material‐dependent covalent functionalization of two‐dimensional TMDC monolayer materials.

## Graphical abstract

PiFM‐led investigation of material‐specific electrochemical aryl diazonium reduction on 2D transition metal dichalcogenide MoSe_2_‐WSe_2_ lateral heterostructure monolayers, with mechanisms explained by molecular dynamics simulations.

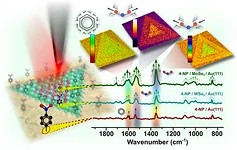

## 1 Introduction

Transition metal dichalcogenides (TMDCs) represent a highly studied class of two‐dimensional (2D) materials with the general formula MX_2_, where M is a transition metal and X a chalcogen. Their layered crystal structure consists of stacked, atomically thin sheets held together by van der Waals forces. Among several approaches for isolating single layers, mechanical or liquid exfoliation and chemical vapor deposition (CVD) are most common, the latter is preferred for producing high‐purity, large‐domain monolayers in a scalable manner [1]. Notably, CVD is an accessible method capable of fabricating seamless, single‐crystal in‐plane TMDC heterostructures, which remain unattainable by exfoliation [2].

CVD‐grown monolayers are ideal substrates for scanning probe microscopy (SPM) due to their atomically flat and smooth surfaces. Typical growth supports are dielectric materials such as mica, sapphire, or SiO_2_/Si. Although TMDCs can be grown on conductive substrates, the high‐temperature CVD environment often induces parasitic reactions that degrade substrate morphology and electronic properties. TMDCs and their thickness are commonly identified by Raman (RS)/photoluminescence (PL) spectroscopy and atomic force microscopy (AFM). Their exceptional chemical and physical stability under ambient conditions makes TMDC monolayers excellent model surfaces for studying functionalization processes and related signal enhancement effects [3, 4, 5].

In this work, monolayer MoSe_2_, WSe_2_, and MoSe_2_‐WSe_2_ in‐plane heterostructures were synthesized by CVD and subjected to surface functionalization through electrochemical reduction of aryl diazonium salts. Aryl diazonium salts are well‐known for their straightforward synthesis and ability to form covalent surface bonds [6]. While numerous reports describe spontaneous or electrochemical diazonium functionalization of TMDCs, direct chemical evidence confirming that optically observed nanofeatures or that spectroscopic signals correspond precisely to grafted species remains limited. Moreover, many studies have relied on exfoliated TMDCs, which do not accurately represent CVD‐grown materials [7, 8, 9, 10, 11, 12, 13, 14, 15, 16].

More recent efforts have focused on CVD‐grown monolayers or nanoribbons, yet often require complex lithographic fabrication of source/drain electrodes and employ destructive high‐energy electron microscopy, introducing unwanted surface modifications [17, 18]. Furthermore, neither carrier mobility measurements nor energy‐dispersive X‐ray spectroscopy (EDXS) can unambiguously identify the chemical fingerprint of the grafted molecule. Table S1 summarizes previous TMDC‐diazonium grafting studies and their respective technical limitations with respect to confident nanometer localized surface chemical assessments. Collectively, these works underscore the need for a simplified, chemically verified approach to functionalize CVD‐grown TMDCs and confirm covalent bonding at the surface.

Electrochemical functionalization poses an additional challenge: the required transfer of TMDCs from their insulating growth substrates onto conductive electrodes. Conventional wet or dry transfer methods using polymethyl methacrylate (PMMA) or polydimethylsiloxane (PDMS) are laborious and often yield damaged or contaminated surfaces [19, 20]. Here, we developed an improved dry‐transfer protocol by combining a metal‐assisted transfer method with template‐stripped gold fabrication to mold the Au substrate around the TMDC monolayers [21]. This approach yielded clean and contamination‐free TMDC monolayers intimately cast into Au(111) electrodes.

Transferred flakes were characterized using visible‐light microscopy (VLM), RS, PL, AFM, Kelvin probe force microscopy (KPFM) and tip‐enhanced Raman spectroscopy (TERS). Knowingly, TERS has been a useful technique applicable to the nanoscale characterization of TMDCs [22, 23, 24]. Using TERS, nanometer resolved images of MoSe_2_‐WSe_2_ vertical‐lateral and in‐plane heterostructure nanodomains and heterojunctions are provided in this work. In addition, a thorough spectroscopic analysis enabled accurate differentiation between the vertically stacked heterobilayer and the lateral nanoheterojunctions. Chemical modifications of the flakes were closely examined by photo‐induced force microscopy (PiFM). In the same manner that far‐field Raman and Infrared spectroscopies offer complementary vibrational data, TERS and PiFM provide a synergistic, nanoscale characterization of the interface, capturing both the polarizable and dipolar molecular transitions at the same order of spatial resolutions. PiFM is a modern infrared spectroscopy technique coupled with AFM, enabling sub‐diffraction chemical mapping with absorption‐like contrast [25, 26, 27, 28]. It provides a high signal‐to‐noise ratio, and down to monolayer sensitivity, particularly on enhancing substrates such as Au or TMDCs [29, 30, 31, 32, 33]. Unlike other AFM‐IR techniques relying mainly on photothermal expansion, the PiFM detection scheme uses a combination of both photo‐induced dipole‐dipole interactions and photothermal expansion, achieving superior sensitivity, with high spatial and chemical resolution [25, 34, 35, 36, 37, 38]. In this study, PiFM measurements were performed using a quantum cascade laser (QCL) tunable from 770 to 1910 cm^−1^, enabling visualization of nitro (‐NO_2_), carboxylate (‐COO), ester (‐COOR), and aromatic (‐Ph) vibrational modes. Comparative AFM and PiFM imaging before and after grafting provided nanoscale chemical maps and spectra, allowing material‐specific reactivity comparisons between monolayer MoSe_2_ and WSe_2_, and within their in‐plane heterostructures.

To complement the experimental observations and provide molecular‐level insight into the surface functionalization mechanism, we employed classical molecular dynamics (MD) simulations. MD numerically integrates Newton's equations of motion for a system of interacting atoms, enabling the direct observation of structural, dynamic, and thermodynamic properties with atomistic resolution [39]. Using well‐parameterized nonbonded force fields, MD can capture the influence of van der Waals and electrostatic interactions and solvent structuring in governing interfacial phenomena [40]. In this work, we modeled MoSe_2_ and WSe_2_ monolayers in aqueous environments containing the 4‐nitrobenzenediazonium ion (4‐NBD^+^) together with explicit counterions (Cl^−^) to maintain charge neutrality. This cation corresponds to the electrochemically relevant precursor of the grafting species prior to reduction. The simulations reproduced the non‐covalent adsorption state under ambient aqueous conditions and quantified differences in interfacial energetics, hydration behavior, and molecular mobility between the two TMDC surfaces, thereby providing a mechanistic rationale for the distinct grafting behavior observed experimentally.

MD investigations on related MoSe_2_‐ and WSe_2_‐based systems further illustrate the importance of atomistic‐level analysis. Xu et al. [41] employed nonequilibrium MD to examine coherent, defect‐mediated, and alloy‐like interfaces in MoSe_2_/WSe_2_ and MoSe_2_/MoS_2_ lateral heterostructures, showing that interface type, lattice mismatch accommodation through out‐of‐plane rippling, and atomic‐scale smoothness have a pronounced influence on phonon transport and thermal conductance. These findings emphasize how subtle variations in interfacial structure can markedly affect energy transfer. Similarly, Jaques et al. [42] conducted fully atomistic simulations comparing the mechanical responses of monolayer and few‐layer WSe_2_ and MoSe_2_ on silicon substrates, finding that monolayers exhibit higher friction coefficients and stronger substrate adhesion than multilayers, and that crack propagation is chirality‐dependent, with zig‐zag‐like defects advancing more readily than armchair ones. Collectively, these studies underscore the roles of interfacial structure, adhesion, and mechanical anisotropy in determining the functional behavior of MoSe_2_ and WSe_2_, offering a computational context for understanding their surface reactivity and stability, and highlighting the importance of MD analysis in capturing these effects.

Building on this foundation, our computational study employs atomistic MD simulations to directly probe the adsorption of 4‐NBD^+^ cations on MoSe_2_ and WSe_2_ monolayers in aqueous solution. Through these simulations, we resolve the molecular‐scale interfacial structure, ion distribution, and solvent‐mediated interactions that govern reactivity. This approach provides the atomistic context that complements our experimental PiFM and voltammetry measurements, bridging nanoscale observables with underlying molecular mechanisms. Here, we use classical MD to model the electrochemically relevant precursor state: noncovalent adsorption of 4‐NBD^+^ on rigid 1T‐MoSe_2_ and 1T‐WSe_2_ monolayers in explicit SPC/E water with counterions, using nonbonded interactions and long‐range electrostatics. This approach captures solvent layering, ion distributions, and time‐resolved precursor mobility/retention that govern local reactant availability at the interface. It does not include applied electrode potential, explicit electron transfer, or bond formation, nor does it treat reactive chemistry or defect evolution, thus we do not claim direct kinetics of reduction or grafting. Within these bounds, MD quantifies interfacial energetics, hydration structure, and residence times (via interaction energies, density profiles, and mean‐squared displacements (MSD)), providing mechanistic variables that are otherwise inaccessible.

## 2 Material and Methods

### 2.1 Chemical Vapor Deposition (CVD) Growth of Transition Metal Dichalcogenides (TMDCs)

Monolayer MoSe_2_ and WSe_2_ were synthesized using two side‐by‐side tubular furnaces (Mini‐Mite TF55030A, Lindberg Blue, Asheville, NC, USA), each equipped with a shared 4‐inch‐diameter quartz tube. Inside the tube, two ceramic combustion boats were aligned with the heating zones. The upstream boat contained 440 mg of selenium (Se, ACS grade) as the chalcogen precursor, while the downstream boat contained either 50 mg of MoO_3_ or 260 mg of WO_3_ (Sigma Millipore) as the transition‐metal oxide precursor. Several 7 mm × 13 mm diced pieces of 300 nm (wet or dry) thermally oxidized SiO_2_/Si wafers (University Wafer, Boston, MA, USA) were placed face‐down across the width of the metal‐oxide precursor boat.

The quartz tube was sealed between a gas inlet and an exhaust port. For MoSe_2_ growth, the downstream zone was heated to 775°C and the upstream zone to 540°C, for WSe_2_, the respective setpoints were 950°C and 540°C. Upon reaching target temperatures, argon (Ar, 4.8 grade) was introduced at 50 sccm as the carrier gas, along with 5 sccm hydrogen (H_2_, 4.9 grade) for MoSe_2_ or 20 sccm for WSe_2_ as the catalyst gas. Reaction durations were 15 min for MoSe_2_ and 10 min for WSe_2_.

MoSe_2_‐WSe_2_ in‐plane heterostructures were grown under the same conditions (950°C downstream, 20 sccm H_2_), except that MoO_3_ and WO_3_ precursors were premixed in the same combustion boat in the same quantities as described above.

### 2.2 Metal Assisted Dry Transfer and Au(111) Fabrication

TMDC growth wafers were affixed backside‐down onto microscope slides using Kapton tape (Ted Pella) and positioned face‐down inside an in‐house physical vapor deposition (PVD) chamber. A 100 nm‐thick gold (Au) layer was deposited onto the exposed wafer surfaces. Following deposition, the wafers were mounted face‐up onto microscope slides with 5‐minute epoxy and allowed to cure overnight.

Diced microscope slide pieces were then coated with UV‐curable adhesive (NOR61, Norland) and placed adhesive‐side down on the Au‐coated growth wafers. The assemblies were irradiated with a 1300 W, 365 nm UV lamp for 30 min and subsequently cured under ambient conditions for one week. The glass pieces were then gently peeled off, yielding TMDC monolayers conformally cast onto the Au films, which were used directly for subsequent experiments (Figure S1).

The resulting Au surfaces were characterized by spinning X‐ray microdiffraction (XRD, Bruker D8 Advance, Cu Kα, *λ* = 1.5406 Å) and atomic force microscopy (AFM) to confirm the (111) crystallographic orientation of gold and verify the expected surface flatness (Figure S2).

### 2.3 Raman Spectroscopy (RS) and Photoluminescence (PL)

Raman spectroscopy (RS) and photoluminescence (PL) measurements were performed using an InVia confocal Raman microscope (Renishaw, Gloucestershire, UK) equipped with a 532 nm green laser and a 100× objective, yielding ≈ 1 µm spatial resolution and 1000× on‐screen magnification. The laser power was set to 5%, the exposure time to 1 s, and the confocal pinhole was engaged (“in”) to ensure a tightly focused spot.

For RS measurements, 10 accumulations were recorded for TMDCs on their growth wafers and 30 accumulations for TMDCs transferred onto Au(111). PL spectra were acquired under identical conditions, except with a 10 s exposure and a single accumulation. RS and PL mapping of individual TMDC flakes were performed using single accumulations per point.

### 2.4 Visible Light Microscopy (VLM)

Visible‐light microscopy (VLM) images were acquired using a Nikon Eclipse LV150 microscope (Tokyo, Japan) to observe TMDCs on their growth wafers, transferred on Au(111), and after electrochemical grafting on Au(111). Images were collected at magnifications of 100×, 200×, 500×, and 1000×.

### 2.5 Electrochemical Grafting of TMDCs

Electrochemical grafting was performed using a three‐electrode configuration consisting of an Ag/AgCl reference electrode, a graphite counter electrode, and a TMDC/Au(111) working electrode. The electrodes were immersed in an electrochemical cell containing 10 mL of freshly prepared grafting solution (0.5 M H_2_SO_4_ and 10 mM of 4‐nitrobenzenediazonium (4‐NBD) salt or 4‐carboxybenzenediazonium (4‐CBD) salt), which had been deaerated with nitrogen for 10 min.

Linear sweep voltammetry (LSV) was performed using an SP‐200 potentiostat (Bio‐Logic, Seyssinet‐Pariset, France) from +0.8 V to −0.5 V (vs Ag/AgCl) at a scan rate of 100 mV s^−1^. Two consecutive sweeps were recorded, and electroactive surface area (ECSA) blockage was evident on the second scan. Prior to each grafting experiment, control LSVs were performed on a polished 1.6 mm Au rod electrode (BASi) to verify solution reactivity.

Following grafting, the TMDC/Au(111) electrodes were immersed in fresh 0.5 M H_2_SO_4_ for at least 1 h to dissolve and remove weakly physisorbed species from the modified surfaces.

### 2.6 Atomic Force Microscopy (AFM) and Photo‐Induced Force Microscopy (PiFM)

AFM images of the TMDCs were captured before and after the electrochemical functionalization on Au(111) by a MultiMode8 (Bruker, Billerica, MA, USA) and VistaScope (MolecularVista, San‐Jose, CA, USA) at resolutions of 512 × 512 pixels or 256 × 256 pixels with a scan rate of 0.5 or 1 line s^−1^ as in previous works [43]. The probes used for the MultiMode8 acquisitions were aluminum backside coated silicon nitride (Si_3_N_4_) ScanAsyst‐Air‐HR with a nominal 2–12 nm tip radius. Those used for the VistaScope recordings were platinum‐iridium (Pt‐Ir) coated Si_3_N_4_ with a ≈20 nm radius of curvature. The PiFM mid‐IR spectra and multispectral chemical images were obtained with the VistaScope microscope within the range of 770 – 1910 cm^−1^. The measurement parameters were optimized to minimize thermal expansion artifacts, focusing on the near‐field force gradient in sideband mode, most relevant to the chemical mapping of the TMDC‐organic interface.

### 2.7 Kelvin Probe Force Microscopy (KPFM) and Tip‐Enhanced Raman Spectroscopy (TERS)

KPFM and TERS measurements were conducted on a LabRam‐Nano AFM‐Raman system (HORIBA Scientific) with a 785 nm laser. A schematic of the tip‐sample is included in Figure 3d. Excitation and collection of TERS signal was done using the side 100 × 0.7 NA objective (Mitutoyo, Kanagawa, Japan) inclined at 25° to the sample plane. OMNI TERS‐SNC‐Au gold coated cantilever probes (AppNano Scientific, CA, USA) and Lprobe TERS03 (VMicro, Hellemmes, France) were used for both the KPFM and TERS characterization. Laser power past the objective for individual wavelengths was kept at ≈100 – 320 µW (1.75%). The integration time was 200 ms/pixel and TERS maps were 1.8 µm × 1.5 µm resolved at 56 × 72 pixels and 1.8 µm × 5.0 µm resolved at 80 pixels, limiting spatial resolutions to roughly 20 nm/pixel and 60 nm/pixel, respectively. Surface roughness and substrate corrugations can significantly dictate the attainable spatial resolution in near‐field measurements, particularly if surface features exceed the tip‐apex radius of curvature (≈25 – 50 nm) or if they impede on tip‐sample stability. In this study, the substrates utilized were characterized by a predominantly (111) crystallographic orientation with high surface planarity, a configuration well‐documented to facilitate high‐resolution TERS imaging. The fidelity of this approach is supported by recent literature investigating TMDC heterostructures transferred onto Au via metal‐assisted dry transfer, which demonstrated spatial resolutions of at least 50 nm [44]. Our experimental architecture utilizes an analogous gap‐mode configuration, wherein the 2D TMDC is sandwiched between two plasmonic surfaces (the Au(111) substrate and the metal‐coated tip) consistent with previously reported high‐resolution configurations [21]. Given the optimized substrate smoothness and the high degree of plasmonic confinement, the theoretical spatial resolution of our system is expected to be below 20 nm. However, the experimental resolutions reported herein were conservatively limited to ≈20 and ≈60 nm, as constrained by the pixel density and scanning window parameters defined in the experimental protocol.

### 2.8 Molecular Dynamics

To uncover molecular‐scale insights into the electrochemical functionalization of TMDCs, we performed atomistic MD simulations designed to replicate the aqueous environment used experimentally to graft aryl diazonium salts onto TMDC monolayers. These simulations aimed to elucidate how surface chemistry, solvation behavior, and molecular mobility contribute to the selective reactivity observed in voltammetry and PiFM measurements. A summary of the MD setup and protocols is provided in (Table S2).

We modeled monolayers of MoSe_2_ and WSe_2_ in the 1T metallic phase with octahedral coordination, using atomic configurations derived from first‐principles calculations reported in a recent study [45]. This choice represents a metallic, electron‐rich structural limit, consistent with the documented 2H → 1T (or 1T’) phase transformation predictions under strongly reductive conditions [46]. Each TMDC was centered within a cubic simulation box of dimensions 100 × 100 × 100 Å^3^. The monolayers were constructed with lateral dimensions of approximately 50 × 30 Å^2^ and consisted of 341 atoms, with selenium atoms symmetrically distributed above and below the transition metal plane. To reproduce the rigid support of Au(111) electrodes, harmonic spring restraints were applied to the TMDC atoms, suppressing structural deformation while maintaining a realistic interfacial environment. The aqueous environment was modeled using the SPC/E water model, which reproduces bulk‐like solution behavior at the target density of 1.0 g cm^−3^. Water bonds were left unconstrained, therefore a 1 fs timestep was used. Six 4‐NBD^+^ together with six chloride counterions were randomly dispersed throughout the simulation box to maintain charge neutrality. This setup represents the noncovalent adsorption state of the diazonium precursor prior to reduction, thereby focusing on surface affinity rather than covalent grafting chemistry, and corresponds to a highly dilute regime that is standard in MD simulations and sufficient for capturing relative adsorption and hydration interactions. Partial charges for water oxygen (−0.8476 e) and hydrogen (+0.4238 e) atoms were taken from the SPC/E model, while atomic charges for 4‐NBD^+^ were derived from CHELPG electrostatic potential fitting at the HF/6‐31G^*^ level of theory using ORCA [47, 48, 49, 50]. Further details are provided in the Supplementary Information—Section S2 (Figure S3). This approach aligns with prior reports that computed CHELPG charges for aryl diazonium ions in MD simulations [51].

Nonbonded interactions were described using a Lennard‐Jones potential combined with long‐range Coulombic electrostatics. Lennard‐Jones parameters for Mo‐Mo, Se‐Se, and W‐W interactions were taken from the Universal Force Field (UFF) [52], which has been used in previous studies for reproducing the cohesive and interfacial behavior of layered TMDCs [53]. The UFF parametrization was chosen instead of Stillinger‐Weber (SW) potential in production run, as our focus was on non‐reactive adsorption and interfacial energetics, UFF provides a consistent Lennard‐Jones framework across both TMDC atoms and molecular species, while avoiding the bond‐order formalisms inherent to reactive force fields. 4‐NBD^+^ was described by the OPLS‐AA force field [54, 55]. Lorentz‐Berthelot mixing rules were applied to define cross‐interactions between TMDC atoms, water, and solute molecules. Long‐range electrostatics were evaluated using the particle‐particle particle‐mesh (PPPM) Ewald summation [56, 57]. Initial atomic coordinates for MoSe_2_ and WSe_2_ monolayers were obtained from separate relaxations using a SW potential, which has been parametrized in a previous study to reproduce realistic bond lengths for various 2D materials, including 1T‐phase MoSe_2_ and WSe_2_ [58], representative parameters and structural details are provided in Supplementary Information—Section 3 (Table S3). Only the atomic positions were transferred, while velocities were discarded, and each structure was subsequently minimized and equilibrated under the UFF + LJ + Coulomb scheme prior to production runs. All parameters match the original UFF paper exactly [52], because UFF tabulates the well depth *D* and the equilibrium distance R*, we convert to LAMMPS Lennard‐Jones form using ε  =  *D* and σ  = R*/2^1/6^, which preserves both the well depth and the position of the potential minimum. Harmonic spring restraints were applied to TMDC atoms, preventing large‐scale lattice relaxation while allowing interfacial interactions to be sampled realistically [59].

The system was first energy‐minimized using a conjugate‐gradient algorithm and then equilibrated under canonical ensemble (NVT) conditions at 298 K for 10 ns. A Nosé‐Hoover thermostat maintained thermal equilibrium throughout [60, 61]. Simulations were run with a timestep of 1 fs, and atomic positions and velocities were recorded every 1 ps. Properties extracted included time‐resolved interaction energies between TMDCs and 4‐NBD^+^, solvent density layering near the TMDC surface, and MSD of 4‐NBD^+^.

### 2.9 Statistical Analysis and Data Treatment

To quantify the spatial resolution of the PiFM maps, we employed a statistical 10% – 90% intensity rise‐profile analysis across sharp high‐intensity features. The raw intensity maps (*ν* = 1349 cm^−1^) were processed using a custom Python‐based script utilizing a minimal Gaussian filter (*σ* = 0.2) to suppress electronic noise while strictly preserving near‐field sharpness. Automated edge detection and population analysis were performed on 50 sharp local intensity maxima (*n* = 50), where the 10% – 90% transition distances were calculated via linear interpolation of the pixel data to ensure sub‐pixel accuracy. The instrumental uncertainty for topography height measurements was determined to be ± 0.35 nm. This value represents the noise floor of the system, characterized by monitoring the vertical deflection channel (AFM *z*‐height) in real‐time while the tip was engaged in an idle state. This baseline measurement accounts for the combined electronic and mechanical instrumental noise under ambient experimental conditions. All PiFM‐IR spectra and PiFM chemical maps were intensity‐normalized to ensure comparability. To isolate the sample signal, raw PiFM‐IR data collected from the template stripped gold Au(111) substrate were averaged and utilized for background subtraction. The resulting spectra underwent a smoothing routine within the same software environment before being exported for further analysis. To account for residual baseline deviations, a cubic‐spline interpolation based on user‐defined anchor points was employed for final baseline correction. Image processing was done with WSxM or Gwyddion [62]. Spectra arithmetics were done with SurfaceWorks/VistaScan 3.0 (Molecular Vista).

## 3 Results

### 3.1 TMDCs Growth and Characterization

**FIGURE 1:**
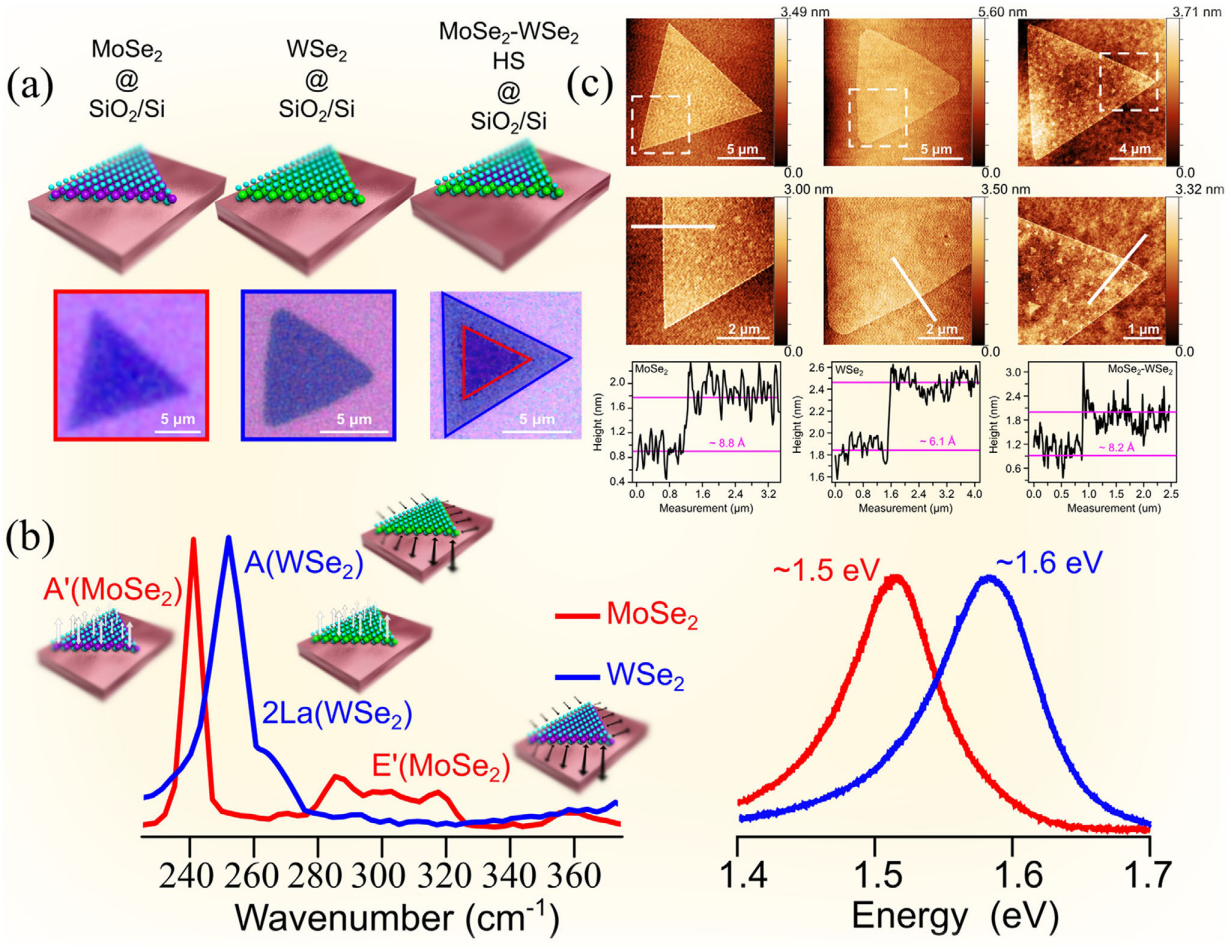
VLM optical images (a), Raman (RS) and photoluminescence (PL) spectra (b), and AFM topography (c) of CVD‐grown TMDCs (MoSe_2_, WSe_2_) and their in‐plane heterostructure (MoSe_2_‐WSe_2_) on a 300 nm SiO_2_/Si substrate. Insets show schematic models of the as‐grown monolayer TMDCs. The red and blue contours in the VLM image of the MoSe_2_‐WSe_2_ heterostructure delineate the MoSe_2_ and WSe_2_ domains, respectively. RS and PL spectra confirmed the chemical identities of the two TMDCs through the A′(MoSe_2_), E’(MoSe_2_), A(WSe_2_), and 2LA(WSe_2_) vibrational modes and their respective direct bandgaps of 1.5 and 1.6 eV. AFM metrology measured typical sub‐nanometer monolayer step heights.

Immediately after CVD growth, the as‐prepared 2D materials were characterized to confirm their chemical composition, structural quality, and monolayer thickness (Figure 1). Visible‐light microscopy (VLM) revealed dark, triangular flakes for MoSe_2_ and WSe_2_ (Figure 1a), consistent with typical monolayer morphologies. The MoSe_2_‐WSe_2_ in‐plane heterostructures exhibited concentric triangular domains with slightly different optical contrast, corresponding to MoSe_2_ at the core and WSe_2_ at the periphery. Raman (RS) and photoluminescence (PL) spectroscopy confirmed this compositional assignment by returning the A′(MoSe_2_), E’(MoSe_2_), A(WSe_2_), and 2LA(WSe_2_) modes at ≈240 cm^−1^, ≈285 cm^−1^, ≈250 cm^−1^, and ≈265 cm^−1^ respectively (Figure 1b). Monolayer thickness was inferred from characteristic spectral features: the A′ out‐of‐plane mode of MoSe_2_ near 240 cm^−1^ and the absence of the 307 cm^−1^ peak in WSe_2_, in agreement with established monolayer signatures [63, 64, 65]. PL measurements yielded direct band gaps of ≈1.5 eV and ≈1.6 eV for MoSe_2_ and WSe_2_, respectively, consistent with literature [66]. The preservation of strong PL emission further verified the monolayer nature of the in‐plane heterostructure, as few‐layer TMDCs exhibit indirect band gaps with quenched PL. Atomic force microscopy (AFM) step‐height analysis between the thermal oxide substrate and flake edges yielded a sub‐nanometer thickness for all three materials, further confirming monolayer format [67] (Figure 1c).

### 3.2 TMDCs Transferred to Au(111)

**FIGURE 2:**
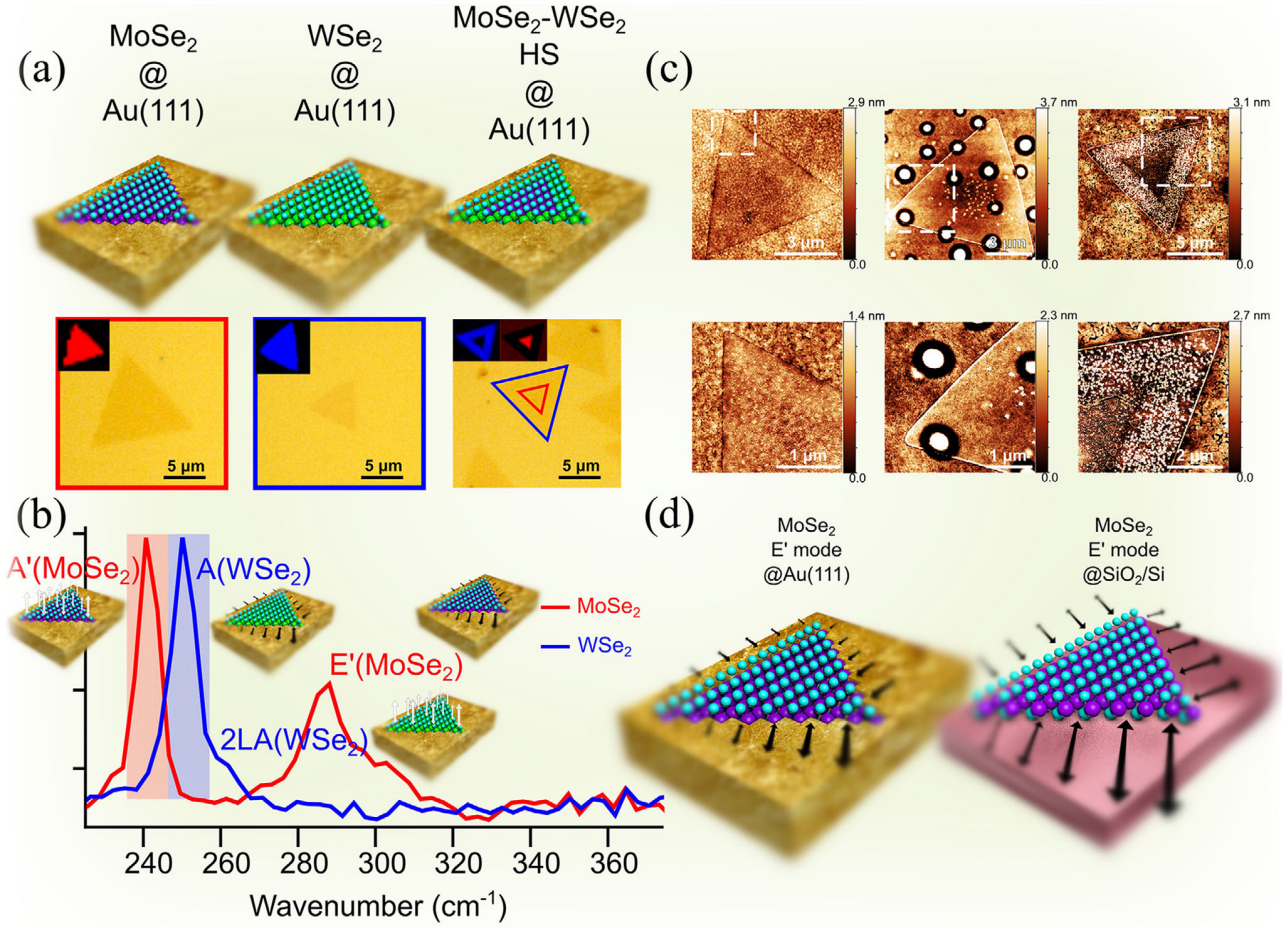
Characterization of TMDC monolayers transferred onto Au(111) substrates by VLM (a), RS (b), and AFM (c). Insets in (a) show RS intensity maps at the spectral shifts highlighted in (b). Schematic models illustrate the TMDC monolayers conformally cast onto the Au(111) surface through metal‐assisted dry transfer, preserving a contamination‐free and damage‐free interface. The red and blue contour lines in the VLM image of the MoSe_2_‐WSe_2_ in‐plane heterostructure delineate the MoSe_2_ and WSe_2_ domains, respectively. Raman spectra reaffirm the chemical identities of MoSe_2_ and WSe_2_ through their characteristic A′, E’, A, and 2LA vibrational modes. AFM imaging could not resolve the monolayer step height, as the transferred monolayers were level with the top surface of the Au(111) substrate; however, RS confirmed the monolayer nature of the flakes. The sharper E’(MoSe_2_) bands observed after transfer are attributed to reduced lateral vibrational dissipation, constrained by the Au(111) substrate tightly molded around the TMDC monolayer structures (d).

The primary limitation of CVD‐grown TMDCs lies in their growth on insulating dielectric substrates, which prevents direct use of their intrinsic electronic properties [21]. Existing transfer protocols toward conductive platforms are typically laborious, complex, and prone to causing structural damage or surface contamination [68, 69]. Here, we integrate a metal‐assisted dry‐transfer method with template‐stripped gold fabrication to relocate monolayer TMDCs onto conductive metallic substrates while fully preserving their as‐grown structural integrity and eliminating adventitious contamination [20, 21, 70, 71]. Post‐transfer, the chemical identity and monolayer nature of the TMDCs were revalidated by VLM and RS (Figure 2a,b).

After transferring the TMDC monolayers onto Au(111), AFM characterization was limited to assessing structural integrity, as the step height no longer provided a reliable measure of layer thickness due to the gold substrate conformally leveling with the flake edges (Figure 2c; Figure S1). Raman spectroscopy (RS) confirmed that the transferred materials remained monolayers. Notably, the E’ mode of MoSe_2_ corresponding to in‐plane intralayer vibrations (Figure 2d), appeared sharper than in spectra acquired from the growth substrate. This effect likely originates from the mechanical constraint imposed by the underlying Au atoms, which stabilize the flake perimeter and restrict in‐plane lattice motion. The transferred TMDCs exhibited enhanced structural robustness, retaining their morphology even after harsh treatments such as ultrasonication, whereas as‐grown flakes readily detached from their dielectric supports. PiFM spectroscopy confirmed the absence of surface contamination (Figure S4), and PL signals were quenched, consistent with a nonradiative quenching of excitons by the Au(111) surface [72].

**FIGURE 3:**
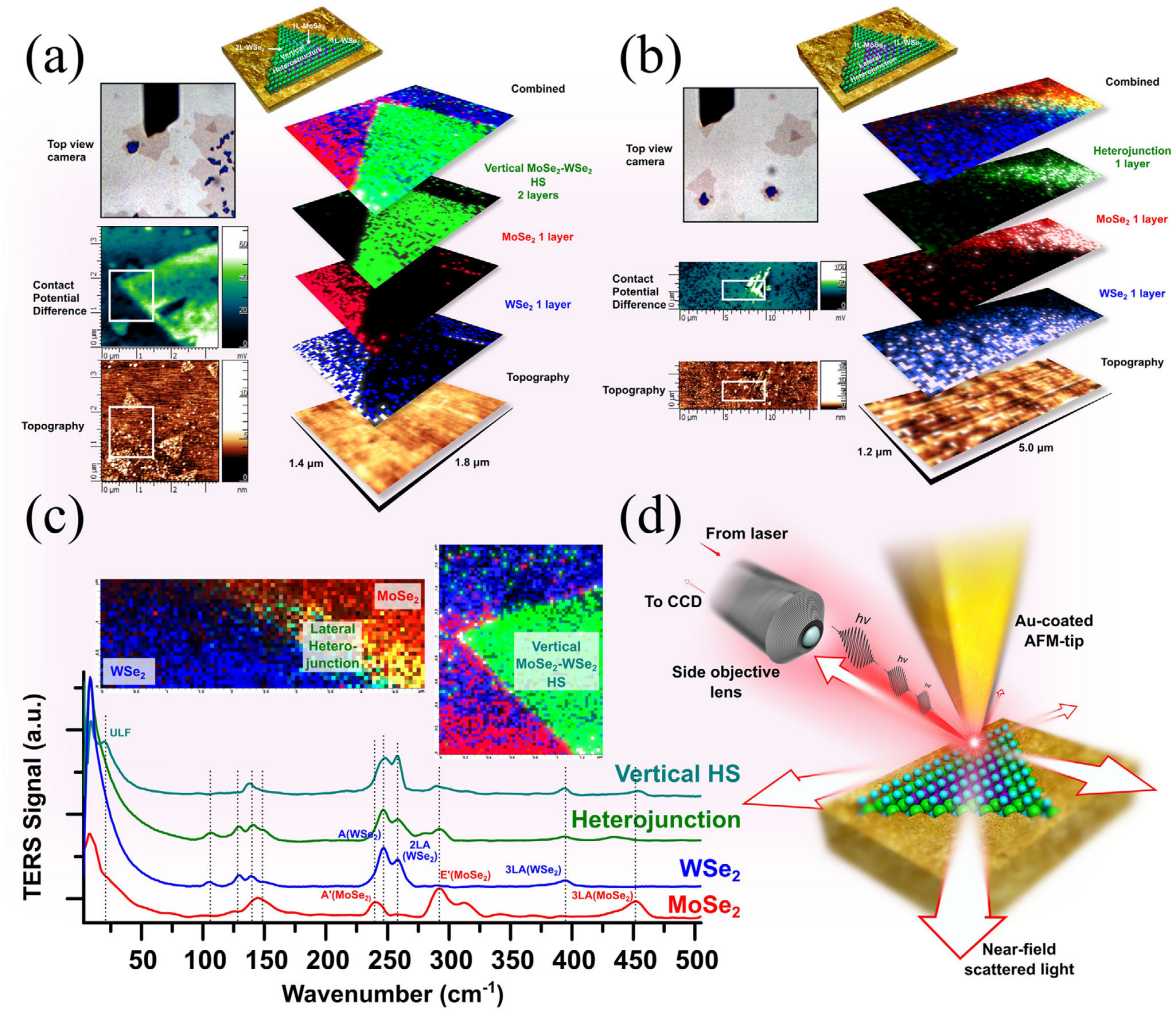
Kelvin probe force microscopy (KPFM) and tip‐enhanced Raman spectroscopy (TERS) nanoscale characterization of heterostructure TMDC monolayers transferred onto Au(111). Magnified KPFM and AFM topography images of the vertical‐lateral MoSe_2_‐WSe_2_ heterostructure (a) and MoSe_2_‐WSe_2_ lateral heterojunction (b). TERS chemical mapping overlay of the heterojunction showing the MoSe_2_ (red), WSe_2_ (blue), transition boundary or bilayer MoSe_2_‐WSe_2_ vertical heterostack (green) and merged domains, acquired with a 785 nm laser at ≈100–320 µW (c). Average TERS near‐field spectra collected from the MoSe_2_, WSe_2_, heterojunction and vertical heterostructure regions indicated by the annotated transparent squares in the merged map insets. The integration time for each spectrum was 200 ms. Schematic representation of the TERS setup probing the MoSe_2_‐WSe_2_ lateral heterostructure (d).

Kelvin probe force microscopy (KPFM) is a nanoscale technique used to measure surface potential. When calibrated with known tip and sample work functions, it provides reliable electronic information about material surfaces, including semiconductor work functions and domain‐specific electronic variations [73]. In conventional AFM imaging, the phase channel often yields strong qualitative contrast by revealing differences in tip‐sample interactions. While this phase contrast is pronounced between a substrate (SiO_2_/Si or Au(111)) and a pure TMDC monolayer, it becomes much weaker when the materials are electronically and mechanically similar, as in lateral or vertical TMDC heterostructures. Alternatively, the contact potential difference (CPD) measured by KPFM is highly sensitive to subtle electronic variations between monolayer, bilayer, and few‐layer regions, providing clear potential‐contrast images [74]. In this study, AFM topography could not distinguish between the MoSe_2_ and WSe_2_ domains of the lateral heterostructures, however, KPFM resolved these regions unambiguously and even revealed nanodomains of bilayer WSe_2_ growing atop the MoSe_2_‐WSe_2_ lateral monolayer heterostructure transition region (Figure 3a). Tip‐enhanced Raman spectroscopy (TERS) is a nanoscale vibrational technique complementary to PiFM. Unlike PiFM, which probes mid‐infrared vibrational modes, TERS enables nanoscale RS through a mechanism closely related to scattering‐type scanning near‐field optical microscopy (sSNOM or nano‐IR). Because neither MoSe_2_ nor WSe_2_ exhibit characteristic mid‐infrared vibrational signatures, PiFM cannot identify their chemical composition, whereas TERS can detect the same Raman modes observed using far‐field confocal RS. While TERS mapping of various monolayer/few‐layer TMDCs and their vertical/lateral heterostructures has been documented on substrates such as hBN, SiO_2_/Si, and Au [21, 44, 63, 74, 75, 76, 77, 78, 79], our contribution provides the first TERS nanoimages and nanospectra of in‐plane MoSe_2_‐WSe_2_ lateral heterostructure alloy junctions directly supported on Au(111), and of their colocalized vertical‐lateral MoSe_2_‐WSe_2_ nanodomains (Figure 3). The TMDC/Au(111) configuration creates a plasmonic gap geometry that enhances the Raman response, enabling high signal‐to‐noise TERS measurements. We performed TERS chemical mapping across the heterojunction and resolved the MoSe_2_ and WSe_2_ domains and a clear alloy transition streak which spanned ≈500 nanometers of lengths with as low as ≈20 – 60 nm spatial precision (Figure 3b). The physical characteristics of the heterojunction are highly dependent on the specific interface architecture. While initial studies on lateral TMDC heterostructures reported atomically sharp interfaces with widths of only a few nanometers [77], more recent investigations have identified broader transition regions. For instance, nanoscale alloyed heterostructures have been shown to exhibit interface widths extending over several hundred nanometers [80]. Furthermore, recent work in 2026 has demonstrated that chemical vapor deposition (CVD) can produce graded alloy interfaces where the junction extends from several hundred nanometers up to the micrometer scale [81]. Given that our experimental CVD parameters closely aligned with those reported in recent literature for graded systems [82], it is highly probable that a gradient alloy heterostructure was formed unintentionally. This provides a robust explanation for the observed wide interface gradient in our samples. Moreover, vertical‐lateral heterostructure nanodomains on several single crystals were also observed (Figure 3a). TERS spectra collected on either side of the junction reproduced the characteristic A′(MoSe_2_) and E’(MoSe_2_) or A(WSe_2_) and 2LA(WSe_2_) Raman bands previously observed at the microscale on SiO_2_/Si and Au(111), confirming the local chemical identities of both domains (Figure 3c). Spectra collected from the alloy zone convoluted both WSe_2_ and MoSe_2_ spectral shifts and contained both 3LA(MoSe_2_) and 3LA(WSe_2_) concurrently, with notable PL. Although the vertical MoSe_2_‐WSe_2_ heterostructure also presented significant photoluminescence and both 3LA(MoSe_2_) and 3LA(WSe_2_) modes, it was differentiable by a distinct ultra low frequency (ULF) interlayer breathing mode noted at ≈25 cm^−1^ [83]. Additionally, in the 85 – 165 cm^−1^ segment, the vertical heterostructure only displayed a single peak, while the lateral heterojunction showed 4 clear deconvoluted peaks: 1 associable to MoSe_2_ and 3 to WSe_2_.

### 3.3 4‐CBD Grafting on MoSe_2_ in Protic Solvent

**FIGURE 4:**
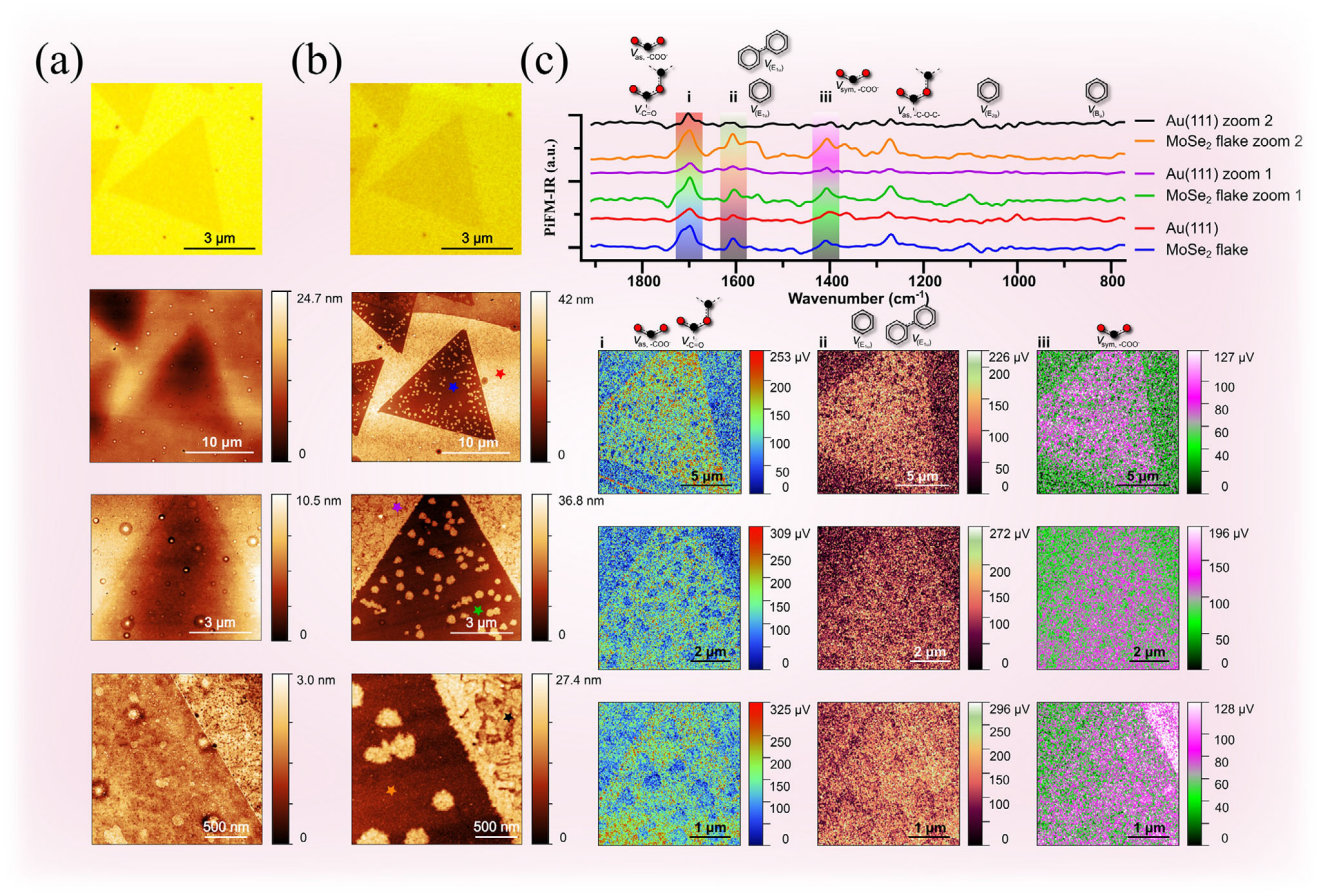
Optical and nanoscale characterization of electrochemically grafted monolayer MoSe_2_. VLM image and AFM topography of a selected monolayer MoSe_2_ flake prior to electrochemical grafting of 4‐CPD (a). Corresponding images after grafting (b). PiFM‐IR spectra recorded at the positions marked by stars in (**b**), with the associated intensity maps shown at the highlighted/annotated wavebands (c). Spectra are color‐coded according to the measurement locations marked by stars. Regions of higher 4‐CP molecules PiFM signal intensity were predominantly located on the MoSe_2_ flake domain, in comparison to the visibly more modified Au(111) substrate surface.

An MoSe_2_ monolayer, identified by RS (Figure S5a) and transferred onto Au(111), was selected for electrochemical (EC) grafting with 4‐carboxybenzenediazonium (4‐CBD). The corresponding EC *I‐V* curves exhibited a pronounced current loss at the reduction peak potential during the second sweep, indicative of electrode surface passivation by covalently attached 4‐carboxyphenyl (4‐CP) films (Figure S5b). Morphological changes before and after grafting were examined by VLM and AFM (Figure 4a, b). A distinct organic overlayer surrounding the MoSe_2_ flake was evident, appearing darker in VLM and more elevated in AFM, while minor surface tears were observed after functionalization. Prior to grafting, the monolayer edge was level with the substrate, post‐grafting, the surrounding region rose by approximately 15 nm, suggesting preferential nanofilm formation on the exposed Au substrate rather than on the flake itself (Figure S6).

PiFM single‐point spectra acquired on both the flake and the adjacent substrate revealed characteristic 4‐CP vibrational resonances, consistent with previous observations [84]. Carboxylic $\nu_{\mathrm{as}(-\mathrm{CO}O^{-})}$ and ester ν_(─COOR)_ stretching bands were observed at ≈1680 – 1700 cm^−1^, while symmetric carboxylic $\nu_{\mathrm{sym}(-\mathrm{CO}O^{-})}$ and asymmetric ether ν_as(─C─O─C─)_ signals were noted at ≈1410 cm^−1^ and 1270 cm^−1^, respectively. $\nu_{\left(E_{1u}\right)}$, $\nu_{\left(E_{2g}\right)}$, and $\nu_{\left(B_{u}\right)}$ aromatic‐ring breathing modes were found at ≈1600 cm^−1^, ≈1000 – 1100 cm^−1^, and ≈820 cm^−1^, respectively. Since PiFM signal intensity correlates with molecular absorption and thus with functional group density [25, 85, 86, 87, 88], the consistently stronger spectral response observed on the flake implied a denser surface concentration of grafted 4‐CP species compared to the substrate. PiFM chemical mapping at the 4‐CP $\nu_{\mathrm{as}(-\mathrm{CO}O^{-})}$/ν_(‐COOR)_, $\nu_{\left(E_{1u}\right)}$, and $\nu_{\mathrm{sym}(-\mathrm{CO}O^{-})}$ vibrational frequencies revealed clear contrast between the MoSe_2_ flake and the substrate, however with consistently stronger intensity pixels localized on the flake basal plane (Figure 4c). Control PiFM maps recorded prior to electrochemical grafting at the same excitation wavenumbers showed no discernible contrast (Figure S5c), confirming that the post‐grafting signal arises exclusively from the covalently attached 4‐CP layer. The formation of aryl diazonium‐derived films is governed by a complex interplay of parameters, including the specific grafting technique, solvent environment, substituent effects, and precursor concentration [89, 90].We distinguished between physisorbed species and chemisorbed layers through three complementary validation methods. First, the modified surfaces were subjected to a rigorous solvent cleaning protocol. Prolonged exposure to media known to solubilize the precursor salts ensured the removal of poorly adsorbed, non‐covalently bonded molecules. Second, electrochemical analysis provided direct evidence of covalent functionalization, the presence of a clear one‐electron transfer reduction peak followed by surface passivation (Figure S5b) confirms the reduction of the diazonium group and subsequent radical‐mediated grafting. Finally, PiFM mid‐infrared nanospectroscopy yielded sharp, consistent peaks characteristic of an oriented and chemically uniform nanofilm [43, 84, 91, 92], rather than the broad, disordered features associated with physisorbed multicomponent aggregates [93]. These findings are consistent with foundational literature regarding the growth of thick polyaryl films. As noted in seminal reviews by Pinson and Bélanger [6, 94], covalently linked aryl films can achieve thicknesses of 100 nm and exceed 1 µm under specific conditions, while retaining a robust, covalently bound, and conjugated polyaryl framework.

### 3.4 4‐NBD Grafting on MoSe_2_, WSe_2_ and MoSe_2_‐WSe_2_ in‐Plane HS Monolayers in Protic Solvent

**FIGURE 5:**
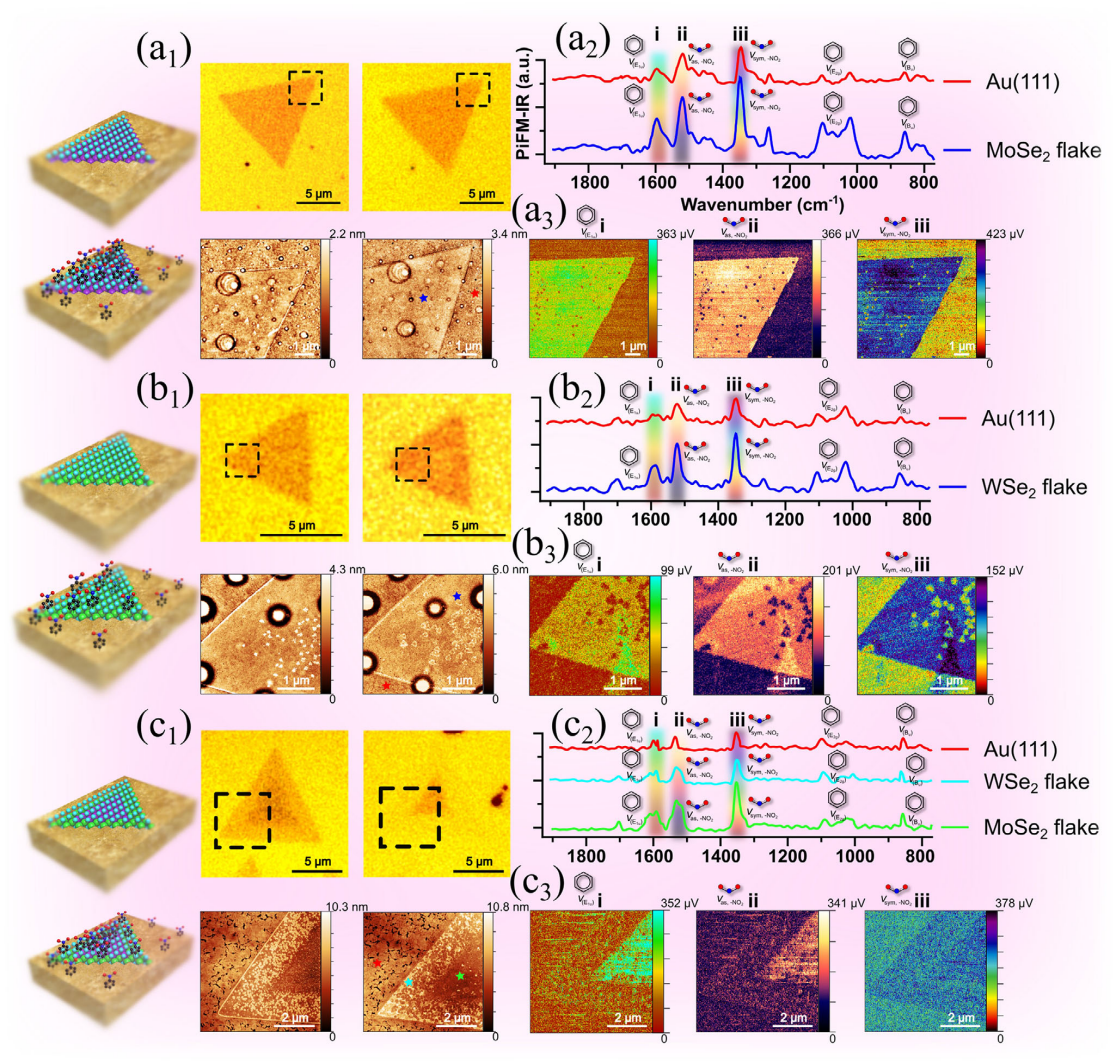
Comparative nanoscale analysis of 4‐NP grafting on TMDC monolayers. VLM and AFM images of monolayer MoSe_2_, WSe_2_, and MoSe_2_‐WSe_2_ lateral heterostructures before and after electrochemical (EC) grafting of 4‐NP (a_1_–c_1_). Single‐pixel PiFM spectra were collected at the positions indicated by a colored star on the corresponding AFM topography images (a_2_–c_2_). PiFM chemical maps were generated at the resonant vibrational modes highlighted in the spectra (a_3_–c_3_). Strong PiFM chemical contrast was observed between 4‐NP‐modified TMDC regions and the surrounding Au(111) substrate (a_2_–a_3_, b_2_, b_3_). For the MoSe_2_‐WSe_2_ in‐plane heterostructure (c_2_, c_3_), the core MoSe_2_ domain exhibited higher 4‐NP signal intensity in both PiFM spectra and mapping compared to the peripheral WSe_2_ region, with both surpassing the gold substrate. These results point to a material‐dependent grafting propensity, with MoSe_2_ displaying the highest reactivity toward 4‐NP functionalization.

When MoSe_2_ was electrochemically functionalized with the reduced 4‐NBD salt, the resulting product differed from that obtained by 4‐CP grafting. While the latter produced a thicker nanofilm on the substrate than on the TMDC monolayer flake, 4‐NP grafting appeared unaffecting the topography before and after the functionalization (Figure 5; Figure S7). Apart from negligible structural deformations caused by the solution‐based grafting and coarse rinsing steps, no significant topographical differences were observed before and after modification. Nonetheless, PiFM single‐pixel spectra revealed the distinct chemical signature attributable to 4‐nitrophenyl (4‐NP) groups both on the flake and on the Au(111) substrate (Figure 5a, _2_, b_2_, and c**_2_**). $\nu_{\mathrm{as}(-N{O_{2}}^{-})}$ and $\nu_{\mathrm{sym}(-N{O_{2}}^{-})}$ could be seen at ≈1520 cm^−1^ and ≈1350 cm^−1^, respectively. While aromatic‐ring breathing modes $\nu_{\left(E_{1u}\right)}$, $\nu_{\left(E_{2g}\right)}$, and $\nu_{\left(B_{u}\right)}$ appeared at ≈1600 cm^−1^, ≈1000 – 1100 cm^−1^, and ≈850 cm^−1^. Although films of similar thickness were detected on both regions, the spectral intensity was again, but this time considerably higher on the flake, suggesting possible signal amplification or interference effects rather than a direct correlation between intensity and surface‐bound molecular quantity. PiFM chemical mapping at the $\nu_{\left(E_{1u}\right)}$, $\nu_{\mathrm{as}(-N{O_{2}}^{-})}$, and $\nu_{\mathrm{sym}(-N{O_{2}}^{-})}$ 4‐NP excitation frequencies provided unprecedented sub‐10 nanometer spatial resolution chemical maps (Figure S8) across the grafted flakes (Figure 5a_3_,b_3_,c_3_; Figure S7). A backside‐exposed folded edge of the MoSe_2_ flake indicated that both sides had been functionalized (Figure 5a_3_; Figure S7a). Even though this portion was not in direct contact with the Au electrode and instead floated in solution, its modification somewhat suggested that the entire flake maintained electrical continuity and conducted electrons during the electrografting, indicating the likely adoption of a conductive (1T) or quasi‐conductive (1T’) phase (Figure S7a). Similar results were obtained for WSe_2_ monolayers functionalized under identical conditions, except for a slightly lower PiFM signal intensity (Figure 5b_3_; Figure S7b). When a MoSe_2_‐WSe_2_ in‐plane heterostructure was subjected to the repeated experiment, 4‐NP signatures were also detected. The resonant peaks were strongest in the central MoSe_2_ region, weaker at the WSe_2_ perimeter, and weakest on the Au(111) substrate (Figure 5c, _2_). PiFM mapping of the 4‐NP modes thus revealed a material‐based trend across the heterostructure: signal intensity decreased from the flake center toward the substrate. It is noteworthy that the metal‐assisted dry transfer process employed herein faithfully preserves the intrinsic morphology of the 2D materials. Consequently, the observed topographical features on the bare flakes are attributed to the source CVD‐grown TMDC substrates, representing defects incurred during the growth phase rather than artifacts of the transfer protocol. Specifically, the spherical features likely represent incomplete nucleation sites [95, 96, 97], while the wrinkles identified across the lateral heterostructure are interpreted as defective basal plane domains or localized strain‐release patterns formed during high‐temperature synthesis [98, 99].

### 3.5 4‐NBD Grafting on MoSe_2_‐WSe_2_ in‐Plane HS Monolayers in Aprotic Solvent

**FIGURE 6:**
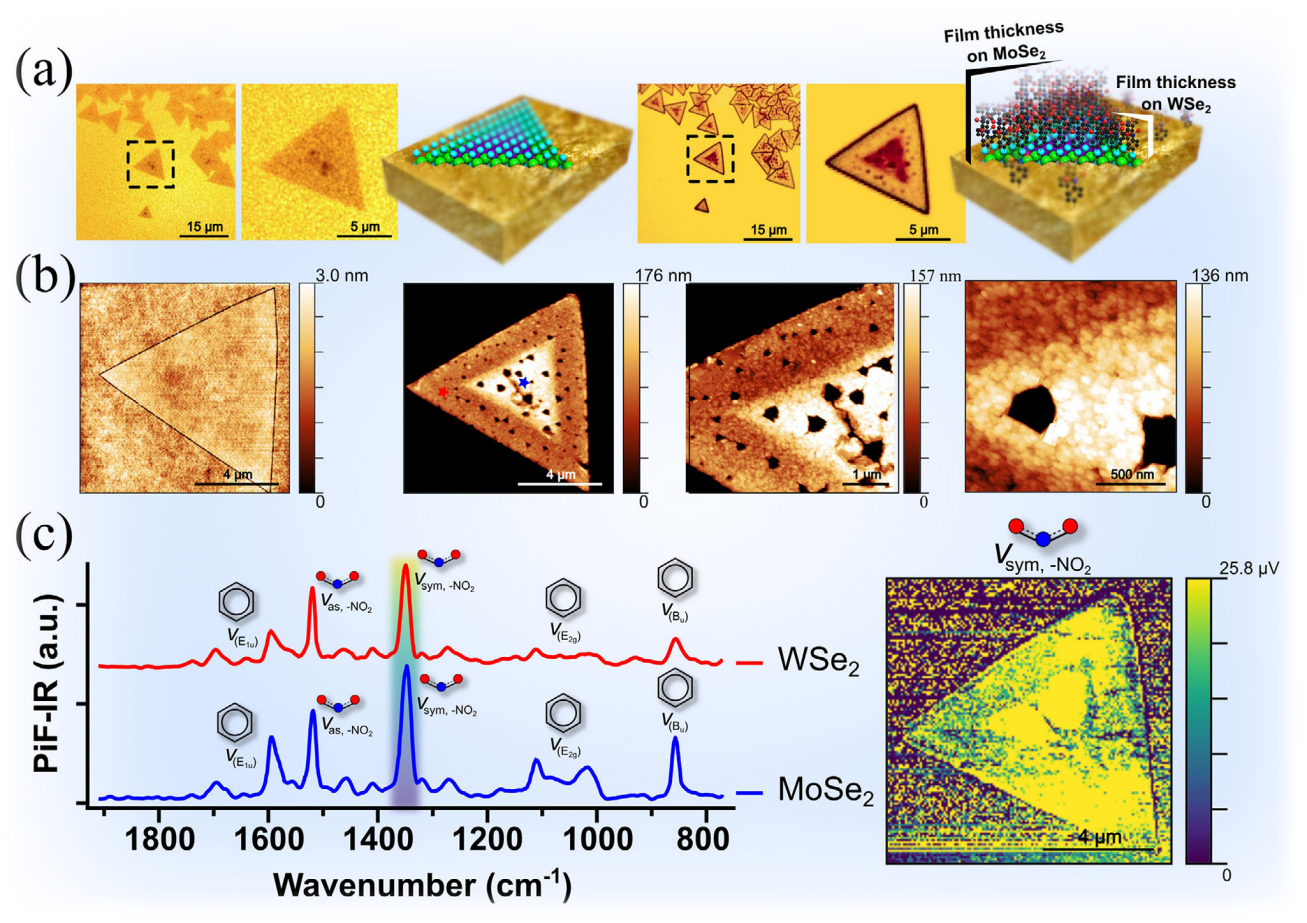
VLM images of the in‐plane MoSe_2_‐WSe_2_ heterostructure monolayer before and after electrochemical grafting with 4‐NP in an aprotic medium (acetonitrile) (a). AFM topography revealed that the flake became substantially thicker on the MoSe_2_ core than on the WSe_2_ periphery after grafting and the ≈500 nm transition alloy heterojunction region can be observed by the gradual decrease in film height (b). PiFM imaging and mid‐infrared surface spectroscopy confirmed the chemical identity of the deposited layer as 4‐NP, despite the signal being saturated because the film thickness on both domains exceeded the depth sensitivity of the PiFM technique (c).

It is well established that grafting performed in protic media typically yields thinner layers approaching monolayer dimensions [100, 101], whereas aprotic solvents, particularly acetonitrile (ACN), favor the formation of thicker organic films [89]. In this work, a monolayer MoSe_2_‐WSe_2_ in‐plane heterostructure was electrochemically functionalized with 4‐NP in ACN (Figure S9a, LSV). After functionalization, the heterostructure became markedly more apparent in VLM images (Figure 6a). AFM revealed drastic topographical changes: the pristine and functionalized surfaces could no longer be corresponded, as a thick organic film had formed across the heterostructure with a maximum thickness of approximately 176 nm (Figure 6b). The inner MoSe_2_ region exhibited a layer ≈65 nm thicker than the peripheral WSe_2_ domain, supporting our previous results in protic conditions and suggesting that MoSe_2_ is more susceptible to diazonium salt electroreduction grafting than WSe_2_. Electrochemical data supported this premise. The reduction (grafting) potential of 4‐NBD on MoSe_2_, WSe_2_, and MoSe_2_‐WSe_2_ HS @ Au(111) revealed a significantly more positive potential for MoSe_2_ (0.51 V vs Ag/AgCl) compared to WSe_2_ (0.37 V vs Ag/AgCl) (Figure S9b,c), implying that grafting on MoSe_2_ is energetically more favorable (−68.2 kJ/mol) than on WSe_2_ (−54.7 kJ/mol). The LSV of the heterostructure displayed two distinct peaks, each corresponding to the characteristic reduction waves of the respective TMDC components. The bare Au(111) substrate showed a single sharp peak at 0.45 V (vs Ag/AgCl), approximately intermediate between the MoSe_2_ and WSe_2_ potentials. Although less obvious in protic media, this same trend was reproduced (Figure S10). Beyond the preferential grafting on the inner MoSe_2_ region, AFM images also revealed scattered triangular pinholes within the grafted films, coinciding with bilayer WSe_2_ nucleation sites. Namely secondary monolayer domains atop the primary layer, indicating that the monolayer regions were more readily functionalized than the bilayer counterparts (Figure S11).

### 3.6 Molecular Dynamics

**FIGURE 7:**
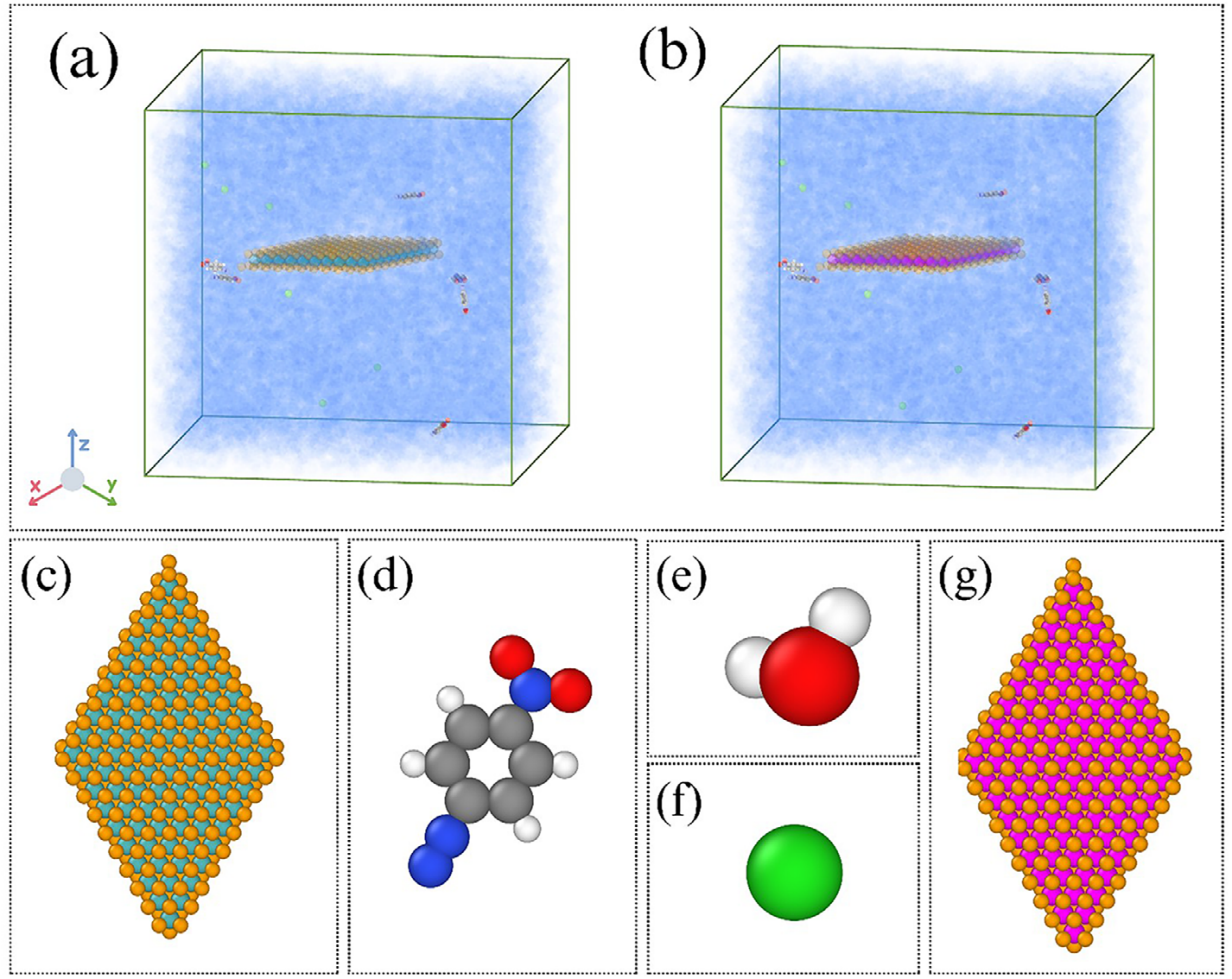
Simulation box containing a MoSe_2_ monolayer immersed in SPC/E water and surrounded by six 4‐NBD^+^ molecules, shown from perspective views (a). Same configuration for WSe_2_ (b). Each TMDC monolayer adopts a 1T metallic phase with octahedral coordination and is held rigid to mimic Au(111)‐supported flakes used in electrochemical experiments. Atomistic structures of the individual components (c–g): MoSe_2_ (c), 4‐NBD^+^ structure (total charge = +1) (d), water molecule (e), chlorine ion (charge = ‐1) (f) and WSe_2_ (g). Simulations represent the electrochemically relevant precursor state (diazonium cations prior to reduction), enabling investigation of solvent structuring and molecular adsorption at the TMDC interface under ambient aqueous conditions. Water molecules are intentionally shown as a blue density cloud for clarity in this visualization.

**FIGURE 8:**
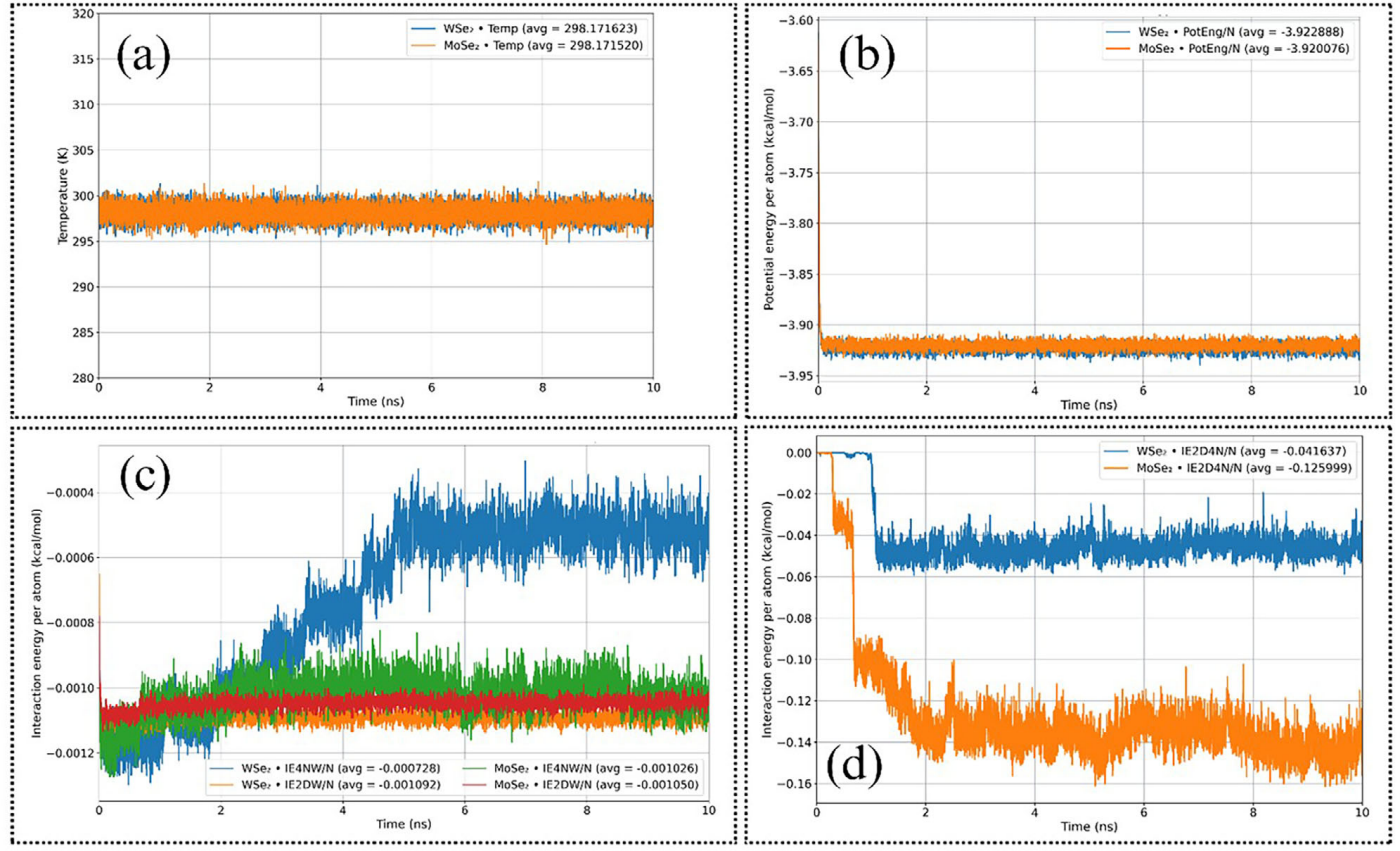
Temporal evolution of thermodynamic and interfacial interaction parameters for MoSe_2_‐water‐4‐NBD^+^ and WSe_2_‐water‐4‐NBD^+^ systems over 10 ns molecular dynamics simulations. Temperature stability confirming equilibrium conditions, with both systems maintained near 298 K (**a**). Potential energy per atom showing rapid stabilization after initial relaxation, with nearly identical cohesive stability for MoSe_2_ and WSe_2_ (**b**). Interaction energy per atom between the 2D layers and either water (IE2DW/N) or 4‐NBD^+^ (IE4NW/N), indicating comparable water affinity but system‐specific differences in 4‐NBD^+^ association (**c**). This suggests that 4‐NBD^+^ retains a more stable hydration environment near MoSe_2_, which could help sustain local solvation and facilitate surface adsorption. Interaction energy per atom between the 2D layers and the combined water + 4‐NBD^+^ (IE2D4N/N), highlighting substantially stronger net adsorption of 4‐NBD^+^ on MoSe_2_ than on WSe_2_ (**d**). Interaction energies shown in this figure are reported per atom (normalizing the group‐group interaction by the number of atoms in the normalizing group). For readability, we also report per‐molecule values for 4‐NBD^+^ in the text, obtained by dividing the total 4‐NBD^+^‐surface interaction by the number of 4‐NBD^+^ molecules. Corresponding per‐molecule 4‐NBD^+^ values are given in the results and in Table S4.

The overall simulation setup, including solvent configurations around MoSe_2_ and WSe_2_, is illustrated in Figure 7. The MD simulations provide a direct comparison of the nonbonded interfacial behavior of MoSe_2_ and WSe_2_ monolayers in the presence of 4‐NBD^+^, Cl^−^ counterions, and water. Figure 8a shows that both systems maintained a stable temperature of ∼298 K over the 10 ns production run, confirming that the trajectories were well equilibrated. In Figure 8b, the potential energy per atom for MoSe_2_ and WSe_2_ quickly stabilized after an initial relaxation, reaching average value of ‐3.92 kcal mol^−1^ atom^−1^. These values are reported on a per‐atom basis for consistency with the simulation outputs, but they correspond to nearly identical cohesive stability of the two TMDC lattices under the modeled conditions. This indicates that any differences in interfacial behavior arise from surface‐specific interactions rather than intrinsic bulk energetics.

The interaction energies presented in Figure 8c are likewise normalized per atom, showing that the surface‐water affinities of the two materials are closely matched. In contrast, the 4‐NBD^+^‐water interaction energies display a more pronounced difference. In the MoSe_2_ system, the average 4‐NBD^+^‐water interaction remains slightly more negative (‐15.4 kcal mol^−1^ per molecule) than in the WSe_2_ system (‐10.93 kcal mol^−1^ per molecule) and stable throughout the simulation, while in the WSe_2_ case the interaction becomes less negative over time, indicating a weakening of hydration in the interfacial region. These trends are consistent with previous DFT‐MD studies reporting more structured interfacial water at MoSe_2_ surfaces [102]. All plots are in kcal mol^−1^ per atom for consistency. The clearest divergence between the two systems appears in Figure 8d, which reports the direct 4‐NBD^+^‐surface interaction energy (IE2D4N) per atom. For MoSe_2_, the average is approximately −0.13 kcal mol^−1^ atom^−1^, about three times more negative than that for WSe_2_ (−0.04 kcal mol^−1^ atom^−1^). When converted to a per‐molecule basis, these values correspond to roughly −9.05 kcal mol^−1^ per 4‐NBD^+^ on MoSe_2_ and −3 kcal mol^−1^ per 4‐NBD^+^ on WSe_2_. In the MoSe_2_ case, the interaction decreases sharply during the first nanosecond, consistent with rapid adsorption of 4‐NBD^+^ molecules onto the surface and then stabilizes at a strongly negative plateau. For WSe_2_, the drop is more modest, and the plateau is less negative, reflecting weaker and less sustained attraction between the surface and 4‐NBD^+^.

Overall, while MoSe_2_ and WSe_2_ are similarly stable in bulk and exhibit nearly the same hydrophilicity under the simulated conditions, MoSe_2_ provides a more favorable surface environment. Specifically, it combines a stronger vdW affinity for 4‐NBD^+^ with a better ability to preserve its hydration shell. Together, these effects increase the interfacial concentration of hydrated 4‐NBD^+^ on MoSe_2_, making reactants more locally available. In practical terms, this may enhance the likelihood of subsequent surface‐driven processes such as electron transfer and covalent grafting [103].

**FIGURE 9:**
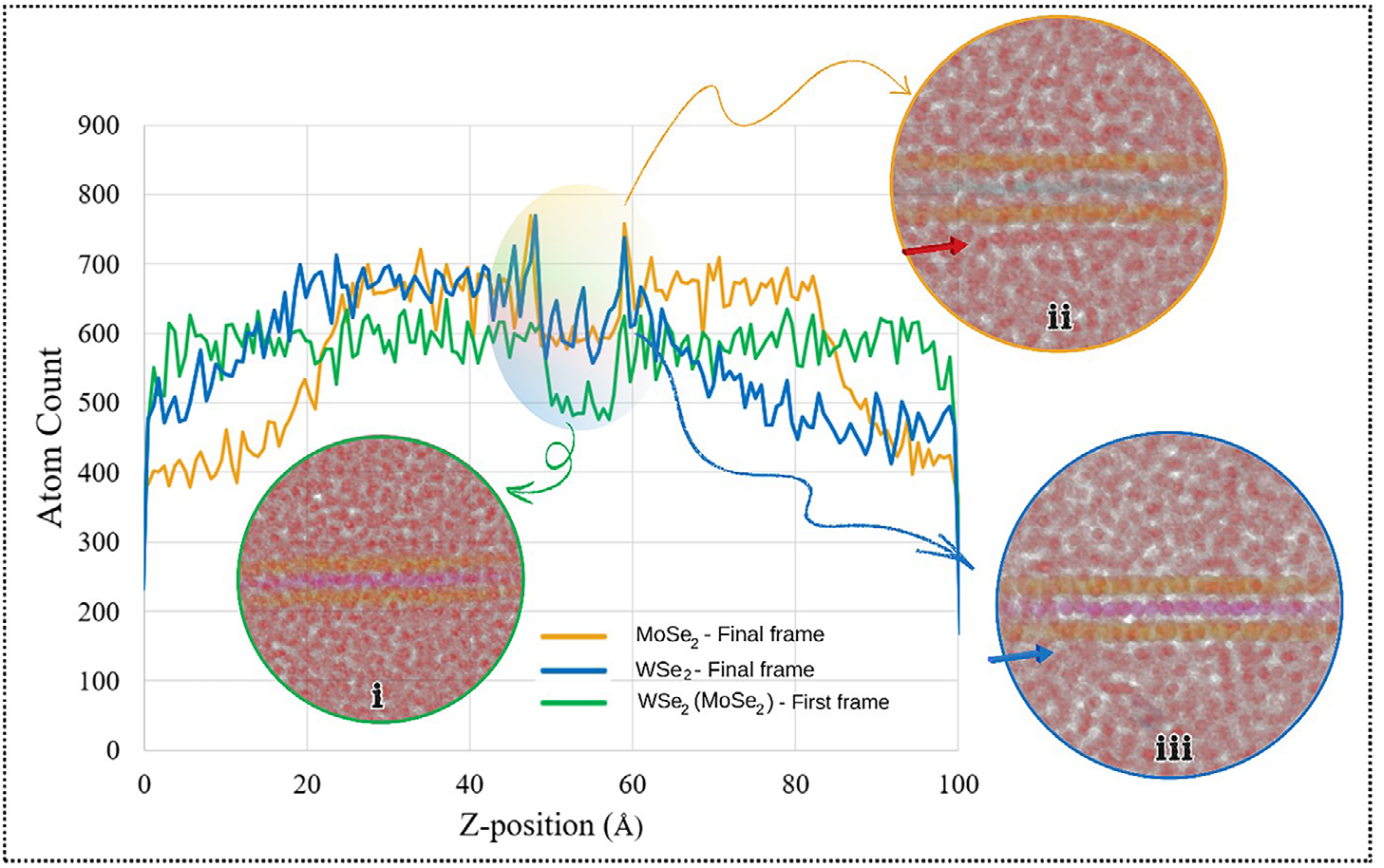
Water density distributions and interfacial snapshots for MoSe_2_ and WSe_2_ systems. Atom count profiles along the Z‐axis are shown for the initial configuration (i: comparable for both systems) and after equilibration (ii: MoSe_2_; iii: WSe_2_). The dip at the center corresponds to the position of the rigid TMDC monolayer. After equilibration, MoSe_2_ exhibits a smooth and symmetric profile with uniform hydration layers, while WSe_2_ displays stronger fluctuations, asymmetry, and local depletion zones. Insets highlight representative snapshots of the hydration environment, illustrating the contrast between ordered interfacial water near MoSe_2_ and disordered structuring near WSe_2_.

To further quantify solvent structuring at the TMDC‐water interface, we analyzed the spatial distribution of water molecules along the Z‐axis (surface normal). Figure 9 shows the water number‐density profiles for the MoSe_2_ and WSe_2_ systems. In all cases, a dip appears at the center of the profile, corresponding to the rigid TMDC monolayer. The initial distribution (green trace) is relatively featureless and low‐density, reflecting the random placement of water molecules before equilibration. After equilibration, the two systems diverge: MoSe_2_ develops a smooth and symmetric profile with uniform hydration layers, whereas WSe_2_ exhibits stronger fluctuations, asymmetric wings, and irregular modulation near the interface. The snapshots inset in Figure 9 reinforce these trends. Initially, both systems display unstructured water near the surface. After equilibration, MoSe_2_ maintains a compact and uniform hydration shell, while WSe_2_ shows depletion zones and uneven layering. These observations are consistent with prior DFT‐MD studies of TMDC‐water interfaces, where Mo‐based dichalcogenides supported more continuous ad‐layer water distributions, whereas WSe_2_ showed patchier coverage and local depletion [104]. Together, the profiles and snapshots demonstrate that MoSe_2_ sustains a more ordered hydration environment, whereas WSe_2_ disrupts interfacial water structuring, conditions that likely contribute to the stronger adsorption of 4‐NBD^+^ observed for MoSe_2_.

These computational observations rationalize the experimental findings that MoSe_2_ exhibits denser grafting coverage in PiFM maps, thicker grafted films in aprotic media, and more positive grafting potentials than WSe_2_. While the present simulations do not capture explicit electron transfer or chemical reactivity, the stronger nonbonded affinity of MoSe_2_ for 4‐NBD^+^ provides a plausible physical contribution to its superior grafting performance. In contrast, the weaker 4‐NBD^+^‐surface attraction and reduced hydration near WSe_2_ create a less favorable interfacial environment for sustaining high local reactant concentrations, consistent with the lower functionalization efficiency observed experimentally.

**FIGURE 10:**
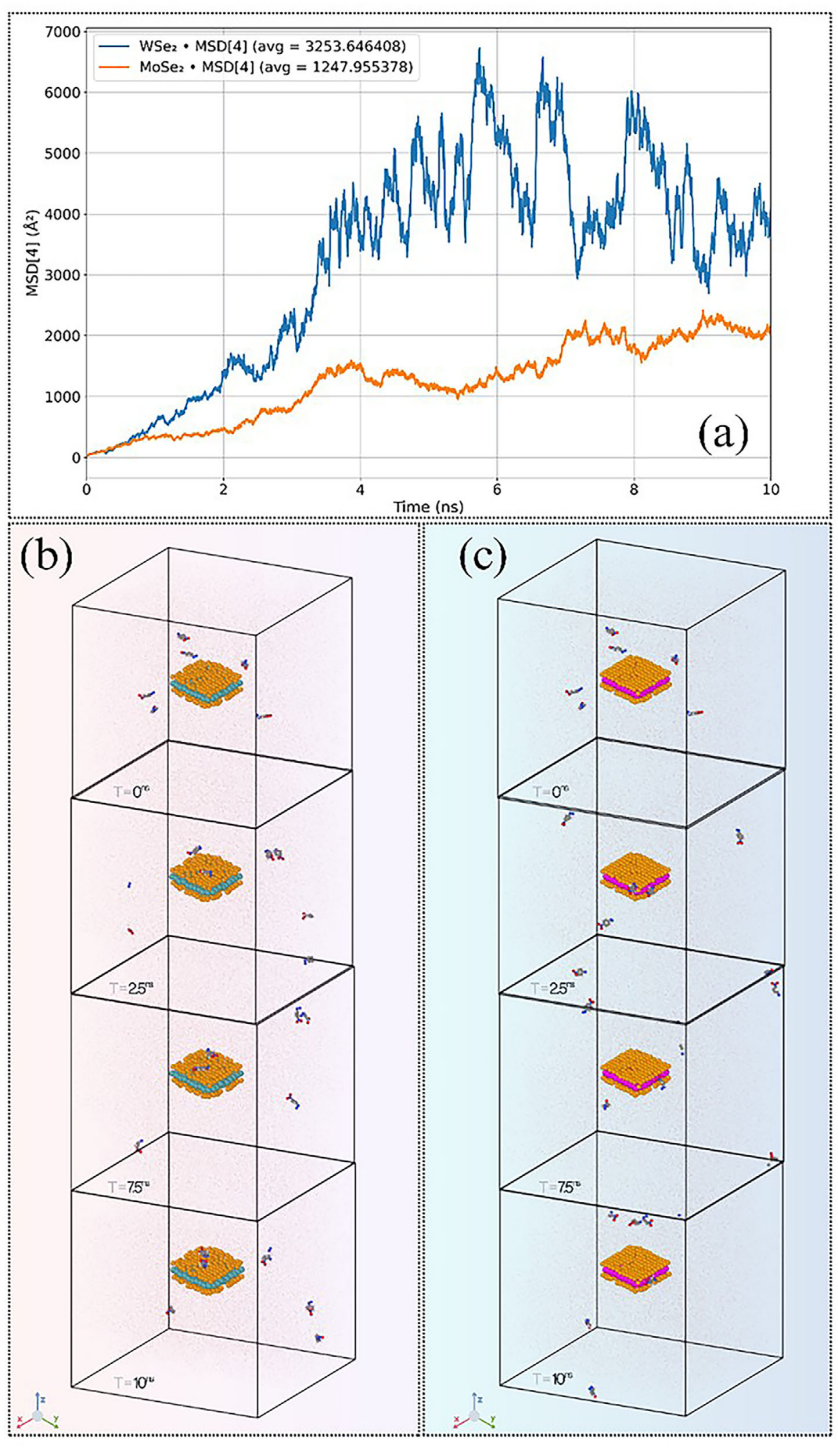
Diffusive and configurational behavior of 4‐NP molecules in MoSe_2_‐water and WSe_2_‐water systems. MSD of 4‐NP molecules in all directions, showing markedly higher mobility in the WSe_2_ system compared to MoSe_2_ (**a**). Time‐resolved snapshots (*t* = 0, 2.5, 7.5, and 10 ns) for MoSe_2_ illustrating persistent surface association and restricted diffusion of 4‐NP (**b**). Corresponding snapshots for WSe_2_ showing greater dispersion of 4‐NP molecules into the aqueous phase, consistent with the higher MSD values (**c**). Water molecules and Cl ions were intentionally removed for clarity in this visualization.

MSD profiles of 4‐NBD^+^ molecules (Figure 10) reveal pronounced differences in mobility. For WSe_2_, the MSD increases rapidly and continuously over the 10 ns trajectory, reaching an average of ≈3250 Å^2^, whereas in MoSe_2_ it remains substantially lower at ≈1250 Å^2^. This separation indicates that MoSe_2_ exerts a stronger physical affinity for 4‐NBD^+^, reducing translational freedom and supporting more persistent surface association. Conversely, the higher MSD values on WSe_2_ reflect weaker net interactions, permitting greater lateral and out‐of‐plane displacements across the simulation cell. In other words, MSD analysis indicates that MoSe_2_ more effectively localizes precursors at its interface, consistent with its stronger adsorption energetics and higher experimental grafting efficiency.

The time‐resolved snapshots (Figure 10b, c) provide direct visual evidence for these mobility trends. In the MoSe_2_ system (Figure 10b), 4‐NBD^+^ molecules exhibit limited displacement from the surface throughout the simulation, with several molecules maintaining proximal contact with the monolayer even after 10 ns. In contrast, the WSe_2_ system (Figure 10c) shows a greater fraction of 4‐NBD^+^ molecules dispersed into the surrounding aqueous phase at intermediate (t = 2.5 ns and t = 7.5 ns) and final time points, consistent with the higher MSD values. Collectively, these results confirm that MoSe_2_ more effectively localizes 4‐NBD^+^ near its surface, enhancing the likelihood of interfacial reaction events via proximity effects.

## 4 Discussion

As an infrared vibrational nanospectroscopy probe, the PiFM compared the surface effects between MoSe_2_ and gold, each attached with 4‐CP, and three different TMDCs functionalized with 4‐NP in protic and aprotic solvents to provide valuable direct nanometer resolved surface chemical information, improbable to achieve with traditional objective‐based spectroscopy microscopes or electron microscopy. WSe_2_ functionalized with 4‐NP showed similar effects to MoSe_2_, with a slightly lesser PiFM intensity. When 4‐CP was grafted on MoSe_2_, a thicker film layer of 4‐CP was seen grafted on the substrate, chemically identified by local spectroscopy. Counterintuitively, the 4‐CP film on the gold substrate, that was ≈15 nm thicker than on the flake, accompanied notably weaker PiFM spectra signals and mapping intensities (Figure 4c; Figure S6). On the other hand, the grafted 4‐NP film on MoSe_2_ was leveled between the substrate and the flake, yet the PiFM spectra signals were again weaker on the gold compared to the flake. This observation prevented straightforward quantitative spectroscopy conclusions. The surface sensitivity of PiFM in sideband coupling mode is primarily governed by the near‐field dipole‐dipole interaction, which exhibits a steep power‐law decay (1/*z* ^4^) [86], as a function of the tip‐sample distance (*z*), consequently, the signal typically saturates for film thicknesses exceeding ≈10 – 20 nm. While the step‐height between the 4‐CP and the MoSe_2_ in this study remains within this interaction volume, achieving a quantitative correlation between surface density and PiFM intensity remains challenging. These limitations are primarily threefold. 1) The layer‐by‐layer reproduction of aryl diazonium films requires nanometer/sub‐nanometer control to ensure consistent density‐to‐signal ratios. 2) Although different PiFM probes yield consistent peak positions, the absolute intensity is highly sensitive to the specific geometry and microscopic protrusions of the tip apex. This tip‐to‐tip variation in the near‐field enhancement factor necessitates complex normalization procedures that often rely on broad approximations. 3) As demonstrated in this work, the dielectric environment of the substrate significantly influences the spectroscopic response. Paradoxically, despite the 4‐CP layer appearing approximately 15 nm thicker on the Au(111) substrate, the PiFM signal was markedly stronger on the MoSe_2_ basal plane, suggesting that substrate‐induced enhancement can outweigh volume density‐based signal contributions. The results of this comparison led to three possible explanations: i) the known optical surface enhancement properties of TMDCs are superior to those of gold (as a flat Au(111) substrate) [105]. ii) Given the much narrower width of the grafted molecules relative to the PiFM and to the AFM physical probe tip‐apexes, the topography was unable to detect that nanofilm packing was superior on the flake compared to the substrate. Or iii) a hybrid effect involving both parameters. The PiFM spectroscopical images probably depicted a more realistic film packing due to its greater resolving power using a sharp localized near‐field extruding from the probe tip‐apex (Figure S12). The latter explanations are the most physically plausible and point toward a substrate‐governed reactivity intrinsic to the materials. As a nanochemical resolved and surface topography method, PiFM provided information about the EC grafting preference between gold and different TMDC materials, supported by voltamperometric analyses. When 4‐NP films were deposited on MoSe_2_ and WSe_2_, the results showed similar properties. Although both materials hosted the grafting material apparently alike, all signals at each 4‐NP resonant mode were stronger on MoSe_2_ than on WSe_2_. This observation agreed with the previously stated hypotheses: either MoSe_2_ was a better surface enhancer, or more tightly and densely grafted than WSe_2_, or both reasons to differing extents. The results of lateral MoSe_2_‐WSe_2_ HS monolayer subjected to 4‐NP graft for an in situ comparison test of these hypotheses were conducive. Core MoSe_2_ expressed greater signal than peripheral WSe_2_ on the PiFM maps. When switching the solvent from protic to aprotic, the 4‐NP film was alarmingly more visible and thicker on the core of the MoSe_2_‐WSe_2_ HS. Both spectra (on MoSe_2_ and WSe_2_) were saturated in signal because of the surpassed ≈10 – 20 nm PiFM probing depth limit in sideband mode. Being ≈65 nm thicker on the central MoSe_2_ domain critically suggested an increasing order of reactivity toward 4‐NP grafting (MoSe_2_> WSe_2_> gold). Attention was drawn to triangular shaped holes on the grafted 4‐NP film. The position of these holes correlated with bilayer nuclei on the HS monolayer prior to EC functionalization. They were found to be less covered by the 4‐NP film and produced pinholes. This supported that the reactivity of the monolayer was greater than that of the few‐layered TMDCs. An explanation could involve that the temporal transition from the thermodynamically stable semiconductor 2H phase to the metastable metallic 1T (or semi‐metallic 1T’) phase is facilitated by significant electron transfer from the gold substrate during electrically driven reduction [46]. This phase modulation may be a key requirement previously overlooked for the effective covalent functionalization of TMDCs via aryl diazonium electrodeposition, as the metallic phase offers the increased surface reactivity necessary for robust bond formation. Real‐time charge‐injection‐driven phase TMDC engineering remains experimentally unproven and is a promising and timely area for future investigation. Exclusively the monolayer flakes could have afforded phase transformation, while bilayers and few‐layers would have required greater driving force (exfoliation and/or chemical intercalation) and therefore remained in 2H form [106]. A recent literature claiming the greater reactivity of exfoliated monolayers MoS_2_ on gold with diazonium salt grafting in comparison to bilayer and few‐layer flakes is in agreement with the herein findings [11]. In summary, the atomistic MD simulations provide a coherent molecular‐scale picture of the interaction between 4‐NBD^+^ with MoSe_2_ and WSe_2_ monolayers in aqueous environments. By explicitly modeling 4‐NBD^+^ and SPC/E water near rigid TMDC substrates, we reproduced the electrochemically relevant precursor state (prior to reduction and covalent bond formation). The simulations revealed that 4‐NBD^+^ preferentially associates with selenium‐rich surface sites of MoSe_2_, while maintaining a more persistent hydration shell compared to WSe_2_. This combination of stronger surface affinity and stabilized solvation provides a plausible atomistic rationale for the experimentally observed higher grafting efficiency of MoSe_2_, as evidenced by PiFM and voltammetry, even though the present MD model only captures the precursor adsorption state and not the reduction or covalent bond formation. However, our MD results pertain to the protic case and may not directly apply to aprotic media, where dielectric screening and specific solvent‐solute interactions differ.

## 5 Conclusions

This work is a remarkable advancement in the study of TMDCs lateral and vertical‐lateral heterostructures while elucidating the scarcely studied aryl diazonium salt functionalization of CVD grown monolayer TMDCs. Unique in‐plane and vertical‐lateral MoSe_2_‐WSe_2_ heterostructure nanodomain and alloy heterojunction TERS analysis and imaging provided valuable evidence of the identity and format of the grown and metal‐assisted transferred TMDCs. Considerable spectroscopic checkmarks were found to unambiguously differentiate between the two types of heterostructures. Motivated by the premise that a topographical change, bulk spectroscopy signatures, differing carrier mobilities, and X‐ray elemental analysis signals do not necessarily imply successful chemical surface functionalization, we showed with high‐resolution AFM measurements that the grafted layer was practically invisible on the monolayer TMDC's basal plane. However, a high PiFM signal intensity correlated with the mid‐infrared signature of the deposited molecules. We reasoned that the spectral intensities could be a result of the better surface enhancement properties of TMDCs relative to gold, or rather a quantitative effect due to greater grafted surface molecular density on the surface of the TMDCs as compared to gold, or a concomitant influence of both mechanisms, manifesting to differing degrees. More likely inclined toward the latter rationales. The same explanations could account for the difference in spectral intensities observed between MoSe_2_ and WSe_2_. Thus, leading to our principal finding which supports material‐specific grafting preferences: MoSe_2_ was seen more favorably grafted than WSe_2_. Furthermore, we showed that monolayers have greater reactivity toward aryl diazonium salt grafting than do bilayers and probably few‐layers, aligning with similar results found in a recent report on exfoliated MoS_2_^11^. MD simulations revealed that solvent‐TMDC interactions govern the MSD and surface‐proximal abundance of aryl diazonium salts, thereby influencing nanofilm formation preferences across TMDC materials. It is worth investigating further layer‐dependence effects on electrochemical functionalization performance and utilizing TERS to resolve the chemical signature and spatial distribution of electrografted polyaryl films on monolayer TMDCs and their corresponding lateral heterostructures. To the best of our knowledge, this is the first sub‐10 nm display of direct non‐destructive resolved colocalized topographical feature and imaging ascribed to infrared chemical signatures of functional molecules covalently grafted on monolayer TMDCs.

## Author Contributions

**Maziar Jafari** and **Mohamed Siaj**: conceptualization. **Maziar Jafari** and **Seyed Faridedin Rafie**: data curation. **Maziar Jafari** and **Seyed Faridedin Rafie**: formal analysis. **Nidal Abu‐Zahra** and **Mohamed Siaj**: funding acquisition. **Maziar Jafari**, **Amir Khojastehnezhad**, and **Andrey Krayev**: investigation. **Maziar Jafari**, **Seyed Faridedin Rafie**, and **Andrey Krayev**: Methodology. **Amir Khojastehnezhad**, **Nidal Abu‐Zahra**, and **Mohamed Siaj**: project administration. **Amir Khojastehnezhad**, **Nidal Abu‐Zahra**, and **Mohamed Siaj**: resources. **Maziar Jafari**, **Seyed Faridedin Rafie**, and **Andrey Krayev**: software. **Amir Khojastehnezhad**, **Nidal Abu‐Zahra**, and **Mohamed Siaj**: supervision. **Maziar Jafari**, **Seyed Faridedin Rafie**, **Andrey Krayev**, and **Amir Khojastehnezhad**: validation. **Maziar Jafari**, **Amir Khojastehnezhad**, and **Mohamed Siaj**: visualization. **Maziar Jafari** and **Seyed Faridedin Rafie**: writing – original draft. **Maziar Jafari**, **Seyed Faridedin Rafie**, **Andrey Krayev**, and **Amir Khojastehnezhad**: writing – review & editing.

## Conflicts of Interest

The authors declare no conflicts of interest.

## Supporting information




**Supporting File**: smll74424‐sup‐0001‐SuppMat.docx.

## Data Availability

The data that support the findings of this study are available from the corresponding author upon reasonable request.
