# Supplementary material for: Material‐Dependent Functionalization of CVD‐Grown TMDC Monolayers Probed by Vibrational Nanospectroscopy
Source: Small. 2026 Jul 6;22(47):e74424. doi: 10.1002/smll.74424 (PMC13495871; doi:10.1002/smll.74424)
Supplement: Supplementary file 1 [file SMLL-22-e74424-s001.docx]

**Material-Dependent Functionalization of CVD-Grown TMDC Monolayers Probed by Vibrational Nanospectroscopy**

Maziar Jafari ^†^, Amir Khojastehnezhad ^‡^, Seyed Faridedin Rafie^⸶^, Andrey Krayev^⸷^, Nidal Abu-Zahra^⸶^, and Mohamed Siaj ^‡, *^

^†^ *Department of Chemistry, Université du Québec à Montréal (UQAM), 201 Av Du Président-Kennedy, Montréal, Québec H2X 3Y7, Canada*

^‡^ *Department of Chemical Engineering and Biotechnological Engineering, Faculty of Engineering, Université de Sherbrooke, 2500 Blvd de l’Université, Sherbrooke, Quebec J1K 2R1, Canada*

^⸶^ *Materials Science and Engineering Department, University of Wisconsin-Milwaukee, Milwaukee, WI 53211, USA*

^⸷^ *HORIBA Scientific, 359 Bel Marin Keys Blvd., Suite 18, Novato 94949, United States*

^*^ Corresponding author:

Mohamed Siaj (Mohamed.Siaj@USherbrooke.ca)

Supplementary Information

[**Table S1.** Timeline of past efforts to functionalize TMDCs with aryl diazonium salts	S3](#_Toc227351087)

[**Figure S1.** Schematic illustration of the metal-assisted dry transfer of a monolayer TMDC onto a template-stripped gold substrate	S6](#_Toc227351088)

[**Figure S2.** X-ray microdiffraction pattern confirming the monocrystalline Au(111) orientation	S7](#_Toc227351089)

[**Table S2.** Summary of molecular dynamics (MD) simulation setup and analysis protocols	S8](#_Toc227351090)

[**Section S2.** Partial Atomic Charges	S9](#_Toc227351091)

[**Figure S3.** CHELPG-derived partial atomic charges for 4-nitrobenzenediazonium cation (4-NBD⁺)	S10](#_Toc227351092)

[**Table S3.** Representative Stillinger-Weber (SW) potential parameters for 1T-MoSe_2_ and 1T-WSe_2_ in LAMMPS format	S11](#_Toc227351093)

[**Section S3.** Stillinger-Weber (SW) Potential for MoSe_2_ and WSe_2_	S11](#_Toc227351094)

[**Figure S4.** AFM topography and PiFM spectra collected from the indicated regions of MoSe_2_, WSe_2_, and the in-plane MoSe_2_-WSe_2_ heterostructure after transfer onto the Au(111) substrate	S12](#_Toc227351095)

[**Figure S5.** RS spectra and Raman mapping at the A’ mode of MoSe_2_, LSVs during electrochemical grafting of 4-CBD, and control PiFM maps at the annotated wavelengths from Figure 4c of the unmodified MoSe_2_ monolayer flake on Au(111)	S13](#_Toc227351096)

[**Figure S6.** AFM measurements of 4-CP grafted film thickness, taken from holes on the flake basal plane and at the edges	S14](#_Toc227351097)

[**Figure S7.** AFM topography at varying magnifications of bare and 4-NP-grafted monolayer MoSe_2_, WSe_2_, and MoSe_2_-WSe_2_ in-plane heterostructures, alongside PiFM chemical maps at 4-NP infrared excitation modes of the same regions analyzed in Figure 5	S15](#_Toc227351098)

[**Figure S8.** Statistical PiFM spatial resolution analysis on the WSe_2_ chemical map at $\nu_{s\text{ym}\left( \text{-}{\text{N}\text{O}_{\text{2}}}^{\text{-}} \right)}$ 1349 cm^-1^	S16](#_Toc227351099)

[**Figure S9.** LSVs of EC grafting of 4-NBD in ACN on MoSe_2_-WSe_2_ in-plane heterostructure, MoSe_2_, WSe_2_, and bare Au(111), alongside a schematic of the functionalization product in aprotic solution	S17](#_Toc227351100)

[**Figure S10.** LSVs of EC grafting of 4-NP in aqueous solution (0.5 M H_2_SO_4_) on MoSe_2_-WSe_2_ in-plane heterostructure, MoSe_2_, WSe_2_, and bare Au(111), along with a schematic of the functionalization product in aqueous solution	S18](#_Toc227351101)

[**Figure S11.** Electrochemical grafting of monolayer TMDCs produces significantly thicker films than on bilayer TMDCs in aprotic solvent	S19](#_Toc227351102)

[**Table S4.** Time-averaged interaction energies for 4-NBD^+^ with MoSe_2_ and WSe_2_ in water at 298 K over a 10 ns production run	S20](#_Toc227351103)

[**Figure S12.** Schematic comparing the radii of curvature of physical PiFM and AFM probes with the near-field PiFM probe	S21](#_Toc227351104)

Table S1. Timeline of past efforts to functionalize TMDCs with aryl diazonium salts, including the characterization techniques used and associated limitations.

| TMDC Material | Synthesis Method | | Material Format | Aryl Diazonium Salt Grafting Method | | Chemical Characterization Techniques and Limitations | | Report |
| --- | --- | --- | --- | --- | --- | --- | --- | --- |
| MoS_2_ | Chemical exfoliation | | Dispersion/flake | Spontaneous in solution | | FTIR, Raman, XPS spectroscopy of bulk solution, no nanometer spatially localized chemical information | | ^1^ |
| MoS_2_, WS_2_, MoSe_2_ | Chemical exfoliation | | Dispersion/flake | Spontaneous in solution | | XPS of bulk functionalized TMDC film surface, no nanometer spatially localized chemical information | | ^2^ |
| MoS_2_ | Mechanical exfoliation, CVD grown | | Flake | Spontaneous on SiO_2_/Si | | FTIR of unwashed flake solution, XPS of functionalized TMDC bulk film surface. No nanometer spatially localized chemical information | | ^3, 4^ |
| MoS_2_ | Exfoliation | | Dispersion/flake | Spontaneous in solution | | IR, Raman, XPS of bulk solution. No localized chemical information | | ^5^ |
| MoS_2_ | Chemical, mechanical exfoliation | | Dispersion/flake | Spontaneous in solution | | SERS, XPS of bulk sample. No nanometer spatially localized chemical information | | ^6^ |
| WS_2,_ WSe_2_ | CVD grown | | Monolayer flakes | Spontaneous on SiO_2_/Si | | XPS of bulk surface, Raman of micro-flake surface, peak-shifts were observed. No nanometer spatially localized chemical information | | ^7^ |
| MoS_2_ | Mechanical exfoliation | | Monolayer, multilayer flakes | Spontaneous on SiO_2_/Si | | Raman of micro-flake surface, no functionalization peaks observed. No nanometer spatially localized chemical information | | ^8, 9^ |
| MoSe_2_, WS_2_, WSe_2_ | Mechanical exfoliation | | Flakes | Spontaneous on SiO_2_/Si, in solution | | FTIR of bulk solution, XPS of bulk surface. No nanometer spatially localized chemical information | | ^10^ |
| MoS_2_ | CVD grown  and PMMA wet-transfer | | Monolayer, few  layer flakes | Electrochemical on Pt electrode | | ATR-FTIR of monolayer MoS_2_ grafted flake with BrP, Observed S-C Peak and grafted molecule signals. Raman spectroscopy and photoluminescence showed peak shifts. XPS showed bromine functional groups signal. Residual PMMA contaminates surfaces even after tentative removal. While elemental mapping via SEM-EDX provides localized nanometer elemental information, these techniques lack the chemical sensitivity required to resolve molecular identities. Furthermore, the high-energy electron beams inherent to these methods are often destructive to organic adlayers, potentially altering the sample's chemical state | | ^11^ |
| MoS_2_ | CVD grown | | Nanoribbons | Device fabrication and electrochemical on SiO_2_/Si | | EDX spectroscopy and electron microscopy are destructive and cause sample beam damage, which may result in mixed elemental signals or noise. Evidence of localized nanometer elemental information, despite destructive, harsh and non-reusable conditions. Localized elemental information may have been compromised by destructive side-reactions and does not directly link to existing surface molecular signatures | | ^12, 13^ |
| MoS_2_ | CVD grown | | Monolayer, few-layer flakes | Spontaneous on SiO_2_/Si | | XPS of undetermined surface shows little or no differences, Raman spectroscopy does not show grafted molecule peaks, only variations in the TMDC’s relative peak intensities. No nanometer spatially localized chemical information | | ^14-16^ |
| MoS_2_ | Chemical exfoliation | Thin film (unspecified) | | | Spontaneous on transistor device | | Raman spectroscopy and XPS of bulk solution. No nanometer spatially localized chemical information | ^17^ |
| MoS_2_ | Mechanical exfoliation | Monolayer, few-layer flakes | | | Spontaneous on Au(111) | | Raman spectroscopy showed no functionalization peaks, and XPS of bulk surface did not show C-S bond. No nanometer spatially localized chemical information | ^18^ |
| MoS_2_ | Chemical exfoliation | Few-layer/bulk | | | Spontaneous on glass or in bulk-solution | | Bulk Raman spectroscopy and XPS were used to describe 4-NP functionalization. Changes in device chemiresistance after functionalization were noted, although unrelated to direct nanometer spatially localized chemical information | ^19^ |
| MoS_2_ | CVD grown  and PMMA wet-transfer | Monolayer | | | Spontaneous on SiO_2_/Si and on Au | | Residual PMMA contaminates surfaces. Tip-enhanced Raman spectroscopy (TERS) and mapping are nanometer localized techniques, however no nanometer spatially direct chemical and structural colocalizations were demonstrated. XPS showed a change in electronic structure of MoS_2_ and cAFM mapped the surface CPD of the flake on Au. Despite the use of nanometer sensitive vibrational spectroscopy techniques, no direct nanometer spatially localized chemical information resulted. | ^20^ |

Figure S1. Schematic illustration of the metal-assisted dry transfer of a monolayer TMDC onto a template-stripped gold substrate, producing a flat TMDC monolayer conformally aligned with the Au(111) surface.

**Figure S2.** X-ray microdiffraction pattern confirming the monocrystalline Au(111) orientation, accompanied by a 6 µm × 6 µm inset 3D AFM topography image showing the characteristic surface flatness of the template-stripped gold (**a**). Low-magnification 30 µm × 30 µm AFM topography scan of the template-stripped gold surface, exhibiting a root mean square roughness (*R*_q_ = ~0.42 nm) approaching the system noise floor (**b**).

**Table S2.** Summary of molecular dynamics (MD) simulation setup and analysis protocols.

| Category | Details |
| --- | --- |
| MD package | Large-scale Atomic/Molecular Massively Parallel Simulator (LAMMPS) ^21^ |
| Systems simulated | (i) MoSe_2_ (1T phase) monolayer + SPC/E water + six 4-NBD^+^ + six Cl^-^ (ii) WSe_2_ (1T phase) monolayer + SPC/E water + six 4-NBD^+^ + six Cl^-^ |
| TMDC structure | Lateral dimensions: ~50 × 30 Å^2^ slab Atoms: 341 (Mo/W + Se) per monolayer |
| Simulation box | Cubic, 100 × 100 × 100 Å³, periodic boundary conditions (PBC) applied in all directions |
| Total atoms | 90 437 (TMDC slab, SPC/E water, 4-NBD^+^, Cl^-^ counterions) |
| Ensemble & thermostat | NVT at 298.15 K using Nosé-Hoover thermostat (applied to solution region) |
| TMDC constraints | Monolayer atoms, tethered by harmonic restraint (spring/self 20.0) to mimic rigid Au-supported flakes and suppress buckling |
| Simulation length | 10 ns production |
| Time step | 1 fs |
| Trajectory outputs | Atomic coordinates and velocities saved every 1 ps |
| Computed properties | (i) Interaction energies: IE2D4N (TMDC ↔ 4-NBD^+^), IE4NW (4-NBD^+^ ↔ Water), IE2DW (TMDC ↔ Water) (ii) Mean-squared displacement (MSD) of 4-NBD^+^ centers of mass |
| Electrostatics | pair_style lj/cut/coul/long 12.0 10.0 with PPPM electrostatics (accuracy 1.0 × 10^-4^) |
| Partial charges | SPC/E water: O −0.8476e, H +0.4238e 4-NBD^+^: CHELPG ESP fitting at HF/6-31G^*^ (**see Section S2**) Cl^-^: −1.0e |
| LJ parameters | Lorentz-Berthelot for cross terms (*σ* arithmetic, *ε* geometric); implemented via explicit pair-coeff cross-entries where needed; validated against default geometric mixing (differences within statistical uncertainty) |
| Mixing rules | Geometric mixing in LAMMPS by default; explicit Lorentz–Berthelot cross terms may be supplied if required |

**Section S2. Partial Atomic Charges (4-NBD⁺)**

Partial atomic charges for the 4-nitrobenzenediazonium cation (4-NBD^+^) were derived by electrostatic potential (ESP) fitting using the CHELPG scheme, as implemented in ORCA 6.1.0^22-24^. The cation was modeled with a total charge of +1 and singlet multiplicity, without an explicit counterion. Geometry optimization and ESP fitting were carried out in a single step at the HF/6-31G^*^ level with tight convergence criteria. CHELPG charges were computed on ORCA’s default Connolly surface and, by construction, sum exactly to +1.0^25^.

Mulliken populations were inspected for consistency but not employed further, owing to their well-known basis set dependence. The resulting CHELPG charges were directly assigned to 4-NBD^+^ atoms in the MD topology without uniform scaling. Lennard-Jones parameters for the same atoms followed the chosen nonbonded force field; no additional polarization terms were introduced. This HF/6-31G^*^ ESP-fit protocol is widely accepted for deriving transferable, nonreactive MD charges and provides methodological continuity with prior diazonium simulations^26^. For reproducibility, the optimized input geometry and full set of per-atom CHELPG charges are reported in Figure S3. For production MD systems explicitly including 4-NBD^+^ and chloride counterions (Cl^-^) were added at 1:1 stoichiometry to ensure overall charge neutrality under Ewald/PME electrostatic

**Figure S3.** CHELPG-derived partial atomic charges for 4-nitrobenzenediazonium cation (4-NBD⁺), obtained at the HF/6-31G^*^ level.

**Table S3.** Representative Stillinger-Weber (SW) potential parameters for 1T-MoSe_2_ and 1T-WSe_2_ in LAMMPS format, adapted from Jiang and Zhou (IntechOpen, 2017).

| System | Interaction Type | A (eV) | σ (Å) | B (Å⁴) | λ (eV) | cos θ₀ | θ₀ (°) | a | Notes |
| --- | --- | --- | --- | --- | --- | --- | --- | --- | --- |
| Mo–Se | Two-body | 2.201 | 1.017 | 20.463 | – | – | – | 3.276 | Bond term |
| Mo–Se–Se | Three-body | – | 1.017 | – | 95.770 | 0.165 | 80.5 | 3.276 | Angle term |
| W–Se | Two-body | 1.885 | 1.013 | 20.186 | – | – | – | 3.277 | Bond term |
| W–Se–Se | Three-body | – | 1.013 | – | 99.800 | 0.165 | 80.5 | 3.277 | Angle term |

# **Section S3. Stillinger-Weber (SW) Potential for MoSe_2_ and WSe_2_**

Initial atomic coordinates for MoSe_2_ and WSe_2_ monolayers were generated using the Stillinger–Weber (SW) parameterization^27^. This parameter set was fitted to reproduce first-principles lattice constants, bond lengths, and elastic properties of single-layer 1T-MoSe_2_ and 1T-WSe_2_, including their characteristic negative Poisson’s ratios. For 1T-MoSe_2_, the equilibrium lattice constant is *a* = 3.2685 Å with a Mo-Se bond length of *d* = 2.5293 Å. For 1T-WSe_2_, *a* = 3.2574 Å with a W-Se bond length of *d* = 2.5207 Å. In both structures, each metal atom is coordinated to six Se atoms (three above and three below the transition-metal plane). Representative SW parameters in LAMMPS format are shown in Table S3. The full parameter sets, including cutoff radii and higher-order terms, are reported in Tables 251-253 (MoSe_2_) and 323-325 (WSe_2_) of Jiang and Zhou’s study^27^. These were used without modification to generate equilibrated starting structures prior to reparametrization under the UFF + LJ + Coulomb scheme for adsorption simulations.

Figure S4. AFM topography and PiFM spectra collected from the indicated regions of MoSe_2_, WSe_2_, and the in-plane MoSe_2_-WSe_2_ heterostructure after transfer onto the Au(111) substrate, highlighting the preserving, clean, and contamination-free nature of the metal-assisted dry transfer method.

Figure S5. RS spectra and Raman mapping at the A’ mode of MoSe_2_ (a), LSVs during electrochemical grafting of 4-CBD (b), and control PiFM maps at the annotated wavelengths from Figure 4c of the unmodified MoSe_2_ monolayer flake on Au(111) (c), showing no chemical contrast between the Au(111) substrate and the monolayer. These results confirm that the chemical contrast observed after functionalization arises specifically from grafted 4-CP molecules.

Figure S6. AFM measurements of 4-CP grafted film thickness, taken from holes on the flake basal plane and at the edges, indicated that the substrate was coated with a 4-CP film approximately 15 nm thicker than the MoSe_2_ monolayer basal plane.

Figure S7. AFM topography at varying magnifications of bare and 4-NP-grafted monolayer MoSe_2_ (a), WSe_2_ (b), and MoSe_2_-WSe_2_ (c) in-plane heterostructures, alongside PiFM chemical maps at 4-NP infrared excitation modes of the same regions analyzed in Figure 5.

Figure S8. Statistical PiFM spatial resolution analysis on the WSe_2_ chemical map at $\nu_{\text{sym}\left( \text{-}{\text{N}\text{O}_{\text{2}}}^{\text{-}} \right)}$ 1349 cm^-1^. Resolved PiFM intensity map showing high-contrast nanostructures (a). Distribution of 10 - 90% rise distances extracted from *n* = 50 high-intensity features (b). The mean spatial resolution was 10.5 nm ± 2.5 and the best resolved feature was measured at 3.1 nm. Measurement based on the line profile in (a) manually determinized the spatial resolution of the PiFM mapping images at ~8.3 nm (c).

Figure S9. LSVs of EC grafting of 4-NBD in ACN on MoSe_2_-WSe_2_ in-plane heterostructure (a), MoSe_2_ (b), WSe_2_ (c), and bare Au(111) (d), alongside a schematic of the functionalization product in solution. Grafting potentials on MoSe_2_ and WSe_2_ are highlighted in red and blue, respectively. The 4-NBD reduction occurs at a more positive potential on MoSe_2_ than on WSe_2_, indicating a more energetically favorable reduction on the former surface.

Figure S10. LSVs of EC grafting of 4-NP in aqueous solution (0.5 M H_2_SO_4_) on MoSe_2_-WSe_2_ in-plane heterostructure (a), MoSe_2_ (b), WSe_2_ (c), and bare Au(111) (d), along with a schematic of the functionalization product in solution. Grafting potentials on MoSe_2_ and WSe_2_ are highlighted in red and blue, respectively. A similar trend is observed between MoSe_2_ and WSe_2_ in protic media, although the potential difference is less pronounced than in organic media (ACN).

Figure S11. Electrochemical grafting of monolayer TMDCs produces significantly thicker films than on bilayer TMDCs in aprotic solvent. Bilayer nucleation regions are indicated by pink lines on both pristine and 4-NP-modified in-plane MoSe_2_-WSe_2_ heterostructures. These results confirm that monolayers are more susceptible to aryl diazonium salt functionalization than bilayers, and even more so than few-layer samples.

**Table S4.** Time-averaged interaction energies for 4-NBD^+^ with MoSe_2_ and WSe_2_ in water at 298 K over a 10 ns production run.

| System | Quantity | Normalization | Value |
| --- | --- | --- | --- |
| MoSe_2_ | 4-NBD^+^–surface IE | per atom | −0.13 kcal·mol⁻¹·atom⁻¹ |
| MoSe_2_ | 4-NBD^+^–surface IE | per molecule | −9.1 kcal·mol^-1^ |
| WSe_2_ | 4-NBD^+^–surface IE | per atom | −0.04 kcal·mol⁻¹·atom⁻¹ |
| WSe_2_ | 4-NBD^+^–surface IE | per molecule | −3.0 kcal·mol^-1^ |
| MoSe_2_ | 4-NBD^+^–water IE | per molecule | −15.4 kcal·mol^-1^ |
| WSe_2_ | 4-NBD^+^–water IE | per molecule | −10.93 kcal·mol^-1^ |

Figure S12. Schematic comparing the radii of curvature of physical PiFM and AFM probes with the near-field PiFM probe. Grafted molecules exhibit a much narrower width (~3.0 - 0.7 Å) than the lateral resolution limits of conventional PiFM (~20 nm) and AFM (~7 nm) tip apexes. In contrast, the near-field emerging from the PiFM tip apex provides substantially higher resolving power (<<< 7 nm), which explains that PiFM can resolve surface packing more effectively than AFM due to its distinct probe nature and superior lateral resolution.

**References**

[1] Knirsch, K. C.; Berner, N. C.; Nerl, H. C.; Cucinotta, C. S.; Gholamvand, Z.; McEvoy, N.; Wang, Z.; Abramovic, I.; Vecera, P.; Halik, M.; et al. Basal-Plane Functionalization of Chemically Exfoliated Molybdenum Disulfide by Diazonium Salts. *ACS Nano* **2015**, *9* (6), 6018-6030. DOI: 10.1021/acsnano.5b00965.

[2] Voiry, D.; Goswami, A.; Kappera, R.; Silva, C. d. C. C. e.; Kaplan, D.; Fujita, T.; Chen, M.; Asefa, T.; Chhowalla, M. Covalent functionalization of monolayered transition metal dichalcogenides by phase engineering. *Nat. Chem.* **2015**, *7* (1), 45-49. DOI: 10.1038/nchem.2108.

[3] Chu, X. S.; Yousaf, A.; Li, D. O.; Tang, A. A.; Debnath, A.; Ma, D.; Green, A. A.; Santos, E. J. G.; Wang, Q. H. Direct Covalent Chemical Functionalization of Unmodified Two-Dimensional Molybdenum Disulfide. *Chem. Mater.* **2018**, *30* (6), 2112-2128. DOI: 10.1021/acs.chemmater.8b00173.

[4] Wang, Q. H.; Chu, X.; Green, A.; Yousaf, A. Method for functionalizing transition metal dichalcogenides. 2018.

[5] Tuci, G.; Mosconi, D.; Rossin, A.; Luconi, L.; Agnoli, S.; Righetto, M.; Pham-Huu, C.; Ba, H.; Cicchi, S.; Granozzi, G.; Giambastiani, G. Surface Engineering of Chemically Exfoliated MoS2 in a “Click”: How To Generate Versatile Multifunctional Transition Metal Dichalcogenides-Based Platforms. *Chem. Mater.* **2018**, *30* (22), 8257-8269. DOI: 10.1021/acs.chemmater.8b03663.

[6] Er, E.; Hou, H.-L.; Criado, A.; Langer, J.; Möller, M.; Erk, N.; Liz-Marzán, L. M.; Prato, M. High-Yield Preparation of Exfoliated 1T-MoS2 with SERS Activity. *Chem. Mater.* **2019**, *31* (15), 5725-5734. DOI: 10.1021/acs.chemmater.9b01698.

[7] Ji, H. G.; Solís-Fernández, P.; Yoshimura, D.; Maruyama, M.; Endo, T.; Miyata, Y.; Okada, S.; Ago, H. Chemically Tuned p- and n-Type WSe2 Monolayers with High Carrier Mobility for Advanced Electronics. *Adv. Mater.* **2019**, *31* (42), 1903613. DOI: 10.1002/adma.201903613.

[8] Li, D. O.; Chu, X. S.; Wang, Q. H. Reaction Kinetics for the Covalent Functionalization of Two-Dimensional MoS2 by Aryl Diazonium Salts. *Langmuir* **2019**, *35* (17), 5693-5701. DOI: 10.1021/acs.langmuir.8b04288.

[9] Wang, Q. H. (Invited) Chemical Functionalization and Transformation of 2D Layered Chalcogenide Materials. *ECS Meeting Abstracts* **2020**, *MA2020-01* (10), 816. DOI: 10.1149/MA2020-0110816mtgabs.

[10] Li, D. O.; Gilliam, M. S.; Chu, X. S.; Yousaf, A.; Guo, Y.; Green, A. A.; Wang, Q. H. Covalent chemical functionalization of semiconducting layered chalcogenide nanosheets. *Mol. Syst. Des. Eng.* **2019**, *4* (4), 962-973. DOI: 10.1039/C9ME00045C.

[11] Park, Y.; Shin, S.; An, Y.; Ahn, J.-G.; Shin, G.; Ahn, C.; Bang, J.; Baik, J.; Kim, Y.; Jung, J.; Lim, H. Tunable Optical Transition in 2H-MoS2 via Direct Electrochemical Engineering of Vacancy Defects and Surface S–C Bonds. *ACS Appl. Mater. Interfaces* **2020**, *12* (36), 40870-40878. DOI: 10.1021/acsami.0c09096.

[12] Lihter, M.; Graf, M.; Iveković, D.; Zhang, M.; Shen, T.-H.; Zhao, Y.; Macha, M.; Tileli, V.; Radenovic, A. Electrochemical Functionalization of Selectively Addressed MoS2 Nanoribbons for Sensor Device Fabrication. *ACS Appl. Nano Mater.* **2021**, *4* (2), 1076-1084. DOI: 10.1021/acsanm.0c02628.

[13] Maldonado, X. S. Selective electrografting of aryl-diazonium salt on cathodically polarized MoS2 nanoribbons. *MRS Bull.* **2021**, *46* (5), 370-371. DOI: 10.1557/s43577-021-00110-7.

[14] Daukiya, L.; Teyssandier, J.; Eyley, S.; El Kazzi, S.; Rodríguez González, M. C.; Pradhan, B.; Thielemans, W.; Hofkens, J.; De Feyter, S. Covalent functionalization of molybdenum disulfide by chemically activated diazonium salts. *Nanoscale* **2021**, *13* (5), 2972-2981. DOI: 10.1039/D0NR07310E.

[15] De Feyter, S. (Invited) Covalent Functionalization of Surface-Supported Graphene and Transition Metal Dichalcogenides: Chemistry and Nanostructuring. *ECS Meeting Abstracts* **2020**, *MA2020-01* (10), 817. DOI: 10.1149/MA2020-0110817mtgabs.

[16] Mali, K. S.; De Feyter, S. (Invited) Covalent Functionalization of Graphene and MoS2: Chemical Versus Electrochemical Routes. *ECS Meeting Abstracts* **2019**, *MA2019-01* (12), 835. DOI: 10.1149/MA2019-01/12/835.

[17] Lee, H.; Bak, S.; Kim, J.; Lee, H. The effect of the dopant’s reactivity for high-performance 2D MoS2 thin-film transistor. *Nano Res.* **2021**, *14* (1), 198-204. DOI: 10.1007/s12274-020-3068-2.

[18] Liu, Z.; Hawthorne, N.; Wu, F.; Sheehan, N.; Argibay, N.; Curry, J. F.; Batteas, J. D. Reactivity of Diazonium Salts on Single- and Multilayer MoS2 on Au(111). *J. Phys. Chem. C* **2022**, *126* (43), 18266-18274. DOI: 10.1021/acs.jpcc.2c03760.

[19] Saha, D.; Angizi, S.; Darestani-Farahani, M.; Dalmieda, J.; Selvaganapathy, P. R.; Kruse, P. Tuning the Chemical and Mechanical Properties of Conductive MoS2 Thin Films by Surface Modification with Aryl Diazonium Salts. *Langmuir* **2022**, *38* (12), 3666-3675. DOI: 10.1021/acs.langmuir.1c03061.

[20] Lambin, C.; McCarvell, W. M.; Cheng, P.; Lau-Truong, S.; Decorse, P.; Mangeney, C.; Félidj, N.; Lagugné-Labarthet, F. Tuning the Band Gap of MoS2 Single Layer with Aryl Diazonium Salts. *J. Phys. Chem. C* **2025**, *129* (10), 5105-5115. DOI: 10.1021/acs.jpcc.4c08755.

[21] Rafie, S. F.; Abu-Zahra, N.; Sabetvand, R. From thin air to pure water: Enhancing atmospheric water harvesting with molecular dynamics simulations of pristine and porous zirconium-based MOF-801. *J. Mol. Liq.* **2025**, *422*, 126939. DOI: 10.1016/j.molliq.2025.126939.

[22] Chio, C. C.; Yang, Y.; Xia, Y.; Tse, Y.-L. S. Molecular Simulations of Fluid Interfaces. *WIREs Computational Molecular Science* **2025**, *15* (4), e70041. DOI: 10.1002/wcms.70041.

[23] Jaques, Y. M.; Manimunda, P.; Nakanishi, Y.; Susarla, S.; Woellner, C. F.; Bhowmick, S.; Asif, S. A. S.; Galvão, D. S.; Tiwary, C. S.; Ajayan, P. M. Differences in the Mechanical Properties of Monolayer and Multilayer WSe2/MoSe2. *MRS Adv.* **2018**, *3* (6-7), 373-378. DOI: 10.1557/adv.2018.246.

[24] Xu, S.; Wu, P.; Chen, Y. Interfacial thermal conductance in 2D WS2/MoSe2 and MoS2/MoSe2 lateral heterostructures. *Comput. Mater. Sci.* **2024**, *245*, 113282. DOI: 10.1016/j.commatsci.2024.113282.

[25] Hanedar, B. A.; Onbaşlı, M. C. Defect dependent electronic properties of two-dimensional transition metal dichalcogenides (2H, 1T, and 1T′ phases). *Phys. Chem. Chem. Phys.* **2025**, *27* (4), 1809-1818. DOI: 10.1039/D4CP04017A.

[26] Neese, F. An improvement of the resolution of the identity approximation for the formation of the Coulomb matrix. *J. Comput. Chem.* **2003**, *24* (14), 1740-1747. DOI: 10.1002/jcc.10318.

[27] Garcia-Ratés, M.; Neese, F. Effect of the Solute Cavity on the Solvation Energy and its Derivatives within the Framework of the Gaussian Charge Scheme. *J. Comput. Chem.* **2020**, *41* (9), 922-939. DOI: 10.1002/jcc.26139.
